# Formative research to design an implementation strategy for a postpartum hemorrhage initial response treatment bundle (E-MOTIVE): study protocol

**DOI:** 10.1186/s12978-021-01162-3

**Published:** 2021-07-14

**Authors:** Meghan A. Bohren, Fabiana Lorencatto, Arri Coomarasamy, Fernando Althabe, Adam J. Devall, Cherrie Evans, Olufemi T. Oladapo, David Lissauer, Shahinoor Akter, Gillian Forbes, Eleanor Thomas, Hadiza Galadanci, Zahida Qureshi, Sue Fawcus, G. Justus Hofmeyr, Fadhlun Alwy Al-beity, Anuradhani Kasturiratne, Balachandran Kumarendran, Kristie-Marie Mammoliti, Joshua P. Vogel, Ioannis Gallos, Suellen Miller

**Affiliations:** 1 Gender and Women’s Health Unit, Centre for Health Equity, University of Melbourne School of Population and Global Health, 207 Bouverie St, Carlton, VIC 3053 Australia; 2 Centre for Behaviour Change, University College London, London, UK; 3 Tommy’s National Centre for Miscarriage Research, Institute of Metabolism and Systems Research (IMSR), WHO Collaborating Centre for Global Women’s Health Research, University of Birmingham, Mindelsohn Way, Edgbaston, Birmingham, B15 2TG UK; 4 Department of Sexual and Reproductive Health and Research, UNDP/UNFPA/UNICEF/WHO/World Bank Special Programme of Research, Development and Research Training in Human Reproduction (HRP), World Health Organization, Avenue Appia 20, Geneva, Switzerland; 5 Maternal & Newborn Health Unit, Technical Leadership Office, Jhpiego, Johns Hopkins University, 1615 Thames Street, Baltimore, MD 21231 USA; 6 Malawi-Liverpool-Wellcome Trust Research Institute, Queen Elizabeth Central Hospital, College of Medicine, Blantyre, Malawi; 7 Institute of Life Course and Medical Sciences, William Henry Duncan Building, University of Liverpool, Liverpool, UK; 8 Africa Center of Excellence for Population Health and Policy, Bayero University, Kano, Kano Nigeria; 9 Department of Obstetrics and Gynaecology, School of Medicine, University of Nairobi, Kenyatta National Hospital Campus, Old Mbagathi Road, Nairobi, Kenya; 10 Department of Obstetrics and Gynaecology, Grooteschuur Hospital, University of Cape Town, Floor H Old Main Building, Anzio Road, Observatory, Cape Town, South Africa; 11 Department of Obstetrics and Gynaecology, University of Botswana, Notwane Rd, Gaborone, Botswana; 12 University of the Witwatersrand, Amalinda Drive, East London, South Africa; 13 Walter Sisulu University, Amalinda Drive, East London, South Africa; 14 Department of Obstetrics and Gynecology, Muhimbili University of Health and Allied Sciences, United Nation Road, Upanga, Dar es Salaam, Tanzania; 15 Department of Public Health, Faculty of Medicine, University of Kelaniya, 6, Thalagolla Road, Ragama, 11010 Sri Lanka; 16 Department of Community and Family Medicine, Faculty of Medicine, University of Jaffna, Adiyapatham Road, Kokkuvil, Sri Lanka; 17 Maternal, Child and Adolescent Health Program, Burnet Institute, 85 Commercial Rd, Melbourne, VIC 3004 Australia; 18 Department of Obstetrics, Gynecology and Reproductive Sciences, School of Medicine, Bixby Center, Safe Motherhood Program, University of California, San Francisco, 550 16th Street, San Francisco, CA 94158 USA

**Keywords:** Maternal health, Postpartum hemorrhage, Obstetric hemorrhage, Care bundle, Formative research, Maternal mortality, Behavior change, Implementation, Intervention development

## Abstract

**Background:**

Postpartum hemorrhage (PPH) is the leading cause of maternal death worldwide. When PPH occurs, early identification of bleeding and prompt management using evidence-based guidelines, can avert most PPH-related severe morbidities and deaths. However, adherence to the World Health Organization recommended practices remains a critical challenge. A potential solution to inefficient and inconsistent implementation of evidence-based practices is the application of a ‘clinical care bundle’ for PPH management. A clinical care bundle is a set of discrete, evidence-based interventions, administered concurrently, or in rapid succession, to every eligible person, along with teamwork, communication, and cooperation. Once triggered, all bundle components must be delivered. The E-MOTIVE project aims to improve the detection and first response management of PPH through the implementation of the “E-MOTIVE” bundle, which consists of (1) **E**arly PPH detection using a calibrated drape, (2) uterine **M**assage, (3) **O**xytocic drugs, (4) **T**ranexamic acid, (5) **I**ntra **V**enous fluids, and (6) genital tract **E**xamination and escalation when necessary. The objective of this paper is to describe the protocol for the formative phase of the E-MOTIVE project, which aims to design an implementation strategy to support the uptake of this bundle into practice.

**Methods:**

We will use behavior change and implementation science frameworks [e.g. capability, opportunity, motivation and behavior (COM-B) and theoretical domains framework (TDF)] to guide data collection and analysis, in Kenya, Nigeria, South Africa, Sri Lanka, and Tanzania. There are four methodological components: qualitative interviews; surveys; systematic reviews; and design workshops. We will triangulate findings across data sources, participant groups, and countries to explore factors influencing current PPH detection and management, and potentially influencing E-MOTIVE bundle implementation. We will use these findings to develop potential strategies to improve implementation, which will be discussed and agreed with key stakeholders from each country in intervention design workshops.

**Discussion:**

This formative protocol outlines our strategy for the systematic development of the E-MOTIVE implementation strategy. This focus on implementation considers what it would take to support roll-out and implementation of the E-MOTIVE bundle. Our approach therefore aims to maximize internal validity in the trial alongside future scalability, and implementation of the E-MOTIVE bundle in routine practice, if proven to be effective.

*Trial registration:* ClinicalTrials.gov: NCT04341662

**Supplementary Information:**

The online version contains supplementary material available at 10.1186/s12978-021-01162-3.

## Plain language summary

Excessive bleeding after birth is the leading cause of maternal death globally. The World Health Organization (WHO) has recommended several treatment options for bleeding after birth. However, these treatments are not used regularly, or consistently for all women. A key underlying issue is that it is challenging for health workers to identify when women are bleeding too much, because measuring the amount of blood loss is difficult.

Maternal health experts have proposed a new clinical ‘care bundle’ for caring for women with excessive bleeding after birth. A care bundle is a way to group together multiple treatments (e.g. 3–5 treatments). These treatments are then given to the woman at the same time, or one after another in quick succession, and supported by strategies to improve teamwork, communication, and cooperation.

This is a research protocol for the preliminary phase of our study (“E-MOTIVE”), which means that it is a description of what we plan to do and how we plan to do it. The aim of our study is to develop a strategy for how we will test whether the E-MOTIVE bundle works through collaborative activities with midwives and doctors in five countries (Kenya, Nigeria, South Africa, Sri Lanka, and Tanzania) to develop a strategy for how we will test whether the E-MOTIVE bundle works. We plan to do this by conducting interviews and surveys with midwives and doctors, and reviewing other research conducted on PPH to understand what works in different settings. We will discuss our research findings in a workshop, with midwives and doctors in the study countries to co-create a strategy that will work for them, based on their needs and preferences.

**Supplementary Information:**

The online version contains supplementary material available at 10.1186/s12978-021-01162-3.

## Background

In low resource countries, every six minutes a woman in the prime of her life, and often with small children, dies from postpartum hemorrhage (PPH) [1]. PPH, defined as a blood loss of 500 mL or more following childbirth, is the leading cause of maternal death worldwide, accounting for 27% of global maternal deaths and affecting 5% of all live births [2, 3]. Women who survive PPH are also at risk for severe maternal morbidities (organ dysfunctions) and longer-term disabilities [4]. The most common cause of PPH is uterine atony (inadequate contraction of the uterus after birth), and PPH can also result from uterine rupture, retained placental tissue, genital tract trauma (vaginal or cervical lacerations), and maternal coagulation disorders [5].

The World Health Organization (WHO) published recommendations for the prevention and treatment of PPH in 2012 to provide evidence-informed recommendations for preventing and managing PPH [5]. When PPH occurs, early identification of bleeding and prompt management with evidence-based interventions can avert most PPH-related severe morbidities and deaths [5]. However, adherence to the WHO recommended practices for PPH treatment remains a critical challenge [6]. For example, data from several low- and middle-income countries (LMICs) show that most women with PPH do not receive life-saving treatment [7]. Analysis of WHO ‘Carbetocin Hemorrhage Prevention’ (CHAMPION) trial data (29,645 women; 10 countries) shows that only 26% (235/886) of women with a blood loss between 500 and 600 mL received a uterotonic drug for PPH treatment [7]. Alarmingly, even with a blood loss of 1000–1100 mL, only 70% (68/96) of women received a uterotonic drug for the treatment of PPH [7]. Moreover, data from over 100 hospitals in Nigeria, Tanzania, and Kenya show that the real-world PPH detection rates are low (Nigeria 2.2%, Tanzania 2.5%, and Kenya 1.8%) [8, 9]. These facilities typically rely on visual estimation of blood loss, widely recognized as inaccurate, and often resulting in underestimation of volume of blood lost [10] and thus detection of PPH.

This raises the question as to what factors are leading to low adherence to PPH recommendations in clinical practice and how adherence could be increased to improve evidence-based practices and quality of care. An important shift in the current thinking is the development of ‘clinical care bundles’ for PPH management. Clinical care bundles are sets of three to five discrete, evidence-based interventions, which are to be administered concurrently or in rapid succession to every person presenting with a specific diagnosis, with the goal of standardizing and expediting care [6]. Care bundles extend beyond decision-making algorithms or checklists, as care bundles aim to improve clinical practice by integrating discrete clinical interventions to be delivered together (concurrently or in rapid succession). Central to care bundles are teamwork, communication, and cooperation, both among healthcare workers and between healthcare workers, women and their families [6, 11]. Bundle compliance is considered achieved when all actions are completed and recorded [6, 11].

In 2017, WHO facilitated a technical consultation to develop two care bundles of clinical interventions for PPH: the “first response to PPH bundle” and the “response to refractory PPH bundle” [6]. The interventions to be considered for inclusion had to have been previously recommended by WHO in its PPH prevention and management guidelines [5, 12]. The elements in the first response to PPH bundle included uterotonics, intravenous fluids, tranexamic acid (TXA), and uterine massage. As with any clinical bundle, they are intended to be introduced along with supportive elements of advocacy, training, teamwork, communication, respectful care, and use of best clinical practices [6].

These supportive elements are critical components of the bundle: an extensive body of implementation research based in high income countries has identified that passive dissemination of new clinical guidelines, including care bundles, alone is unlikely to result in improved quality of care [13]. Indeed, implementing a new guideline or recommendations in practice will almost always require someone to do something differently. This can involve adopting an entirely new practice, replacing one practice with another, doing more or less of an existing practice, or discontinuing a practice altogether [14]. That is, implementation almost always requires behavior change, often in individual and collective behaviors of healthcare providers, as well as at organization, service delivery, and system levels [15]. Such behaviors are typically complex. This is particularly true of implementing care bundles, which typically involve multiple actors (e.g. healthcare providers), working together to deliver and perform multiple clinical actions concurrently or in rapid succession [16]. These behaviors are likely to be influenced by an equally complex set of interacting individual, socio-cultural, and environmental influences. Therefore, designing interventions to change clinical practice and improve implementation first requires understanding of the influences on current and desired behaviors in the context in which they occur [15, 16].

Previous research exploring influences on implementation of care bundles identified barriers related to: staffing levels, case acuity, lack of awareness, lack of self-efficacy, inappropriate expectations, overloaded or inadequate staff qualified to implement, lack of engagement of staff or management, fear of added work of record keeping, and fear of reprisals for not complying with bundle elements [17–19]. Conversely, factors supporting or enabling implementation of bundles include: perceived sustainability, positive, supportive leadership and champions of the bundle, resources, training, focus on quality of care, teamwork, communication, and including bundle compliance into routine record keeping [17, 18]. However, this evidence is primarily from high-income settings and concerns implementation of care bundles for other clinical areas (e.g. chronic obstructive pulmonary disease, sepsis). There is limited evidence regarding challenges to care bundle implementation in LMICs, particularly in maternal health and emergency settings, which could include factors such as lack of staff with appropriate competencies, lack of essential supplies including medications and overburdened services.

## The E-MOTIVE study

**Fig. 1:**
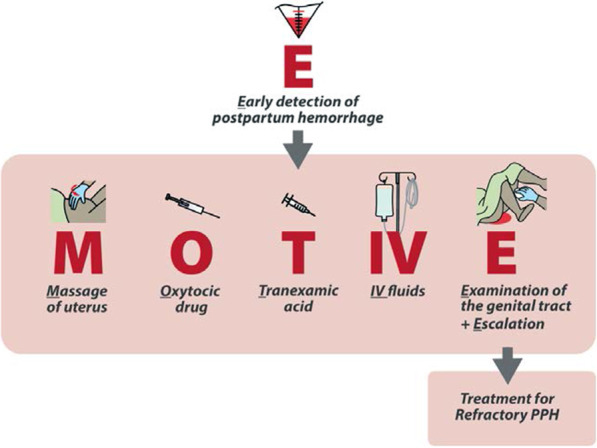
The E-MOTIVE care bundle

**Fig. 2:**
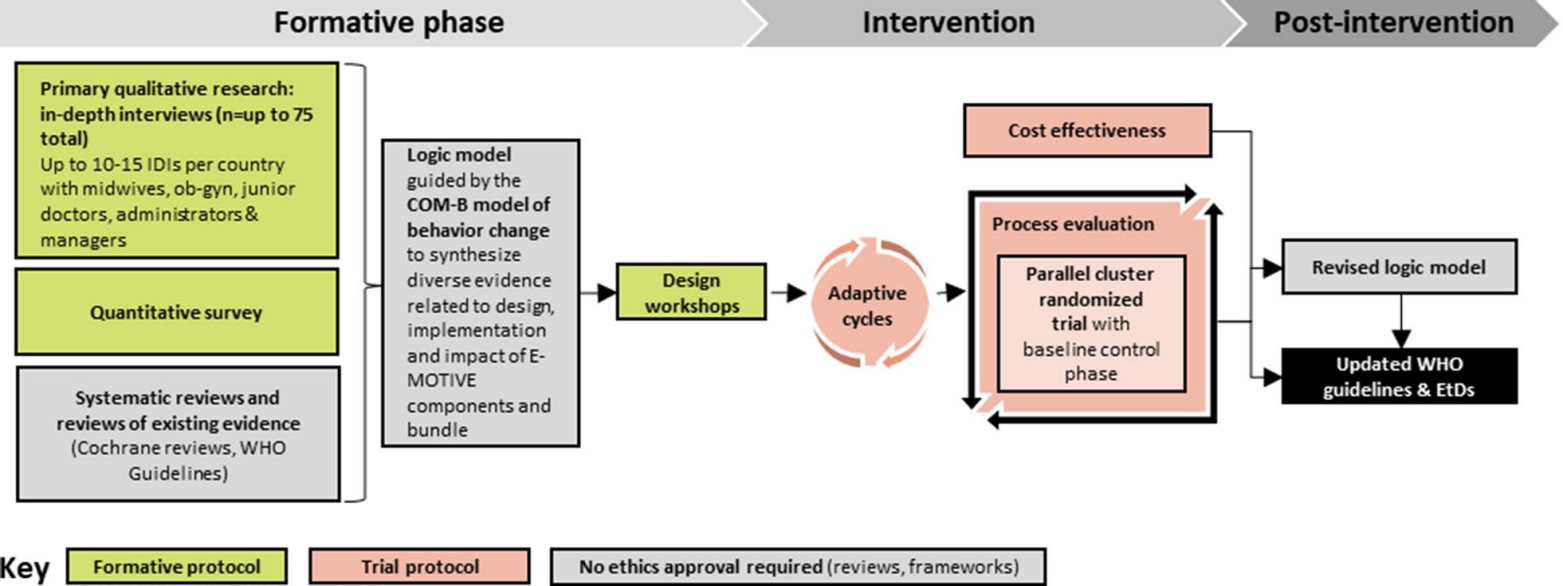
Overview of the E-MOTIVE research projects. The formative components of the project are outlined in this protocol. Subsequent publications will outline the adaptive cycles, parallel cluster randomized trial, process evaluation and cost-effectiveness. *COM-B* model of behavior change referring to capability, opportunity, and motivation; *IDIs* in-depth interviews, *EtD* evidence-to-decision frameworks, *WHO* World Health Organization

The E-MOTIVE program of research aims to improve the detection and first response management of PPH through the implementation of a new care bundle called “E-MOTIVE” (Fig. 1). The E-MOTIVE bundle consists of (1) **E**arly detection of postpartum hemorrhage using an under-buttocks, calibrated blood collection drape, (2) **M**assage of the woman’s uterus, (3) administration of **O**xytocic drugs, (4) administration of **T**ranexamic acid, (5) administration of **I**ntra**V**enous fluids, and (6) **E**xamination of the woman’s genital tract and escalation when necessary. E-MOTIVE will take place in three key phases: a formative phase, intervention phase, and post-intervention phase (Fig. 2). During the intervention phase, a parallel cluster randomized trial with a baseline control phase will take place, along with a mixed-methods process evaluation and cost effectiveness study. In the post-intervention phase, if the E-MOTIVE bundle is effective, clinical guidelines will be updated to reflect new evidence. However, as noted previously, dissemination of the bundle alone is unlikely to result in change in practice. Therefore, alongside the bundle, the E-MOTIVE program of research aims to develop, adapt, and evaluate an implementation strategy to support clinician behavior change and uptake of the bundle in practice.

This is the focus of the formative phase (Phase 1) of the E-MOTIVE program of research, and the focus of this study protocol. E-MOTIVE Phase 1 intervention development will use mixed-methods to understand how PPH is currently managed in the study settings, and the potential barriers and enablers to implementing the E-MOTIVE bundle. This will provide a basis for designing an implementation strategy with healthcare staff at the study sites to support uptake and delivery of the E-MOTIVE bundle during the trial and in wider practice. This will involve a series of workshops (described below) between research staff and facility-based staff in the countries.

We plan to use a mixed-methods approach in the formative research (Fig. 2). In line with best practice guidance for designing and evaluating complex interventions [20], the formative phase will incorporate theory-based intervention development, followed by a small-scale pilot and mixed-methods process evaluation to explore the feasibility and acceptability of the intervention and implementation strategy and potential contextual modifiers. This will enable iterative refinement of the implementation strategy ahead of the definitive trial in Phase 2. This protocol focuses on describing the formative research. A separate protocol will be published for the feasibility pilot study, and the E-MOTIVE trial is registered at ClinicalTrials.gov (NCT04341662).

### Study conceptual frameworks

**Fig. 3:**
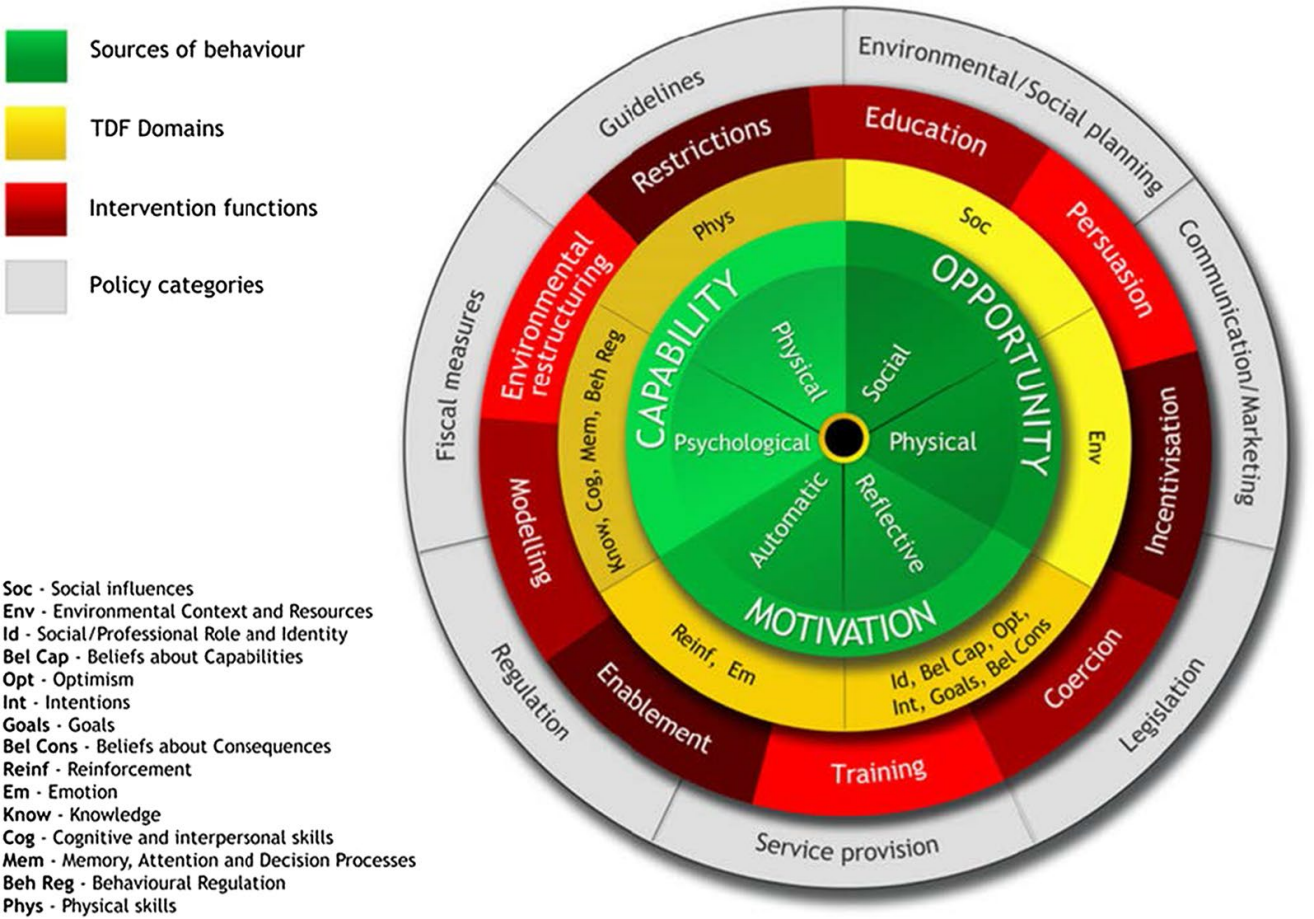
Integrated study conceptual frameworks: COM-B Model, Theoretical Domains Framework, Behavior Change Wheel [13]

There are many overlapping behavior change theories, with limited guidance available for selecting amongst potentially relevant theories [21]. Thus, there have been efforts to synthesize behavior change theories into integrated, overarching theoretical frameworks and models. One such model is the COM-B model [13] and the associated Theoretical Domains Framework (TDF) [22], which posits that in order for a desired **B**ehavior to occur (i.e. clinical practice action), the individual must have the **C**apability, **O**pportunity, and **M**otivation to do so (Fig. 3) [13]. Both frameworks synthesize numerous theories of behavior change into a core set of domains (e.g. factors) representing individual, socio-cultural and environmental influences on behavior. Capability involves factors such as knowledge, skills, decision making, attention, and memory. Opportunity concerns how our environment influences behavior, and includes environmental context factors (e.g. time, access to necessary supplies, resources, staffing, infrastructure) and social context (e.g. practice norms, professional identity, team working, pressure, support, role modelling). Motivation includes internal processes that energize and direct behavior, and factors such as priority, goals, perceived benefits, risk and consequences, emotions, habit, incentives, and threat.

Both frameworks have been widely applied in implementation research to explore barriers and enablers to changing clinical practice behaviors and improving implementation across various clinical contexts (e.g. improving implementation of sepsis care bundles and blood transfusion practice) [15, 19, 23]. A benefit of COM-B and the TDF is that they are mapped to two associated behavioral science frameworks specifying different types of intervention strategies. First, the Behaviour Change Wheel [13] which specifies nine broad intervention types (e.g. education, modelling, incentivization, environmental restructuring). Second, the Behaviour Change Technique Taxonomy, which specifies 93 more granular techniques for changing behavior (e.g. goal-setting, feedback, self-monitoring) [24]. There are published matrices pairing domains of COM-B or TDF with interventions in the Behaviour Change Wheel and taxonomy [25–27], thereby linking influences on behavior to fit-for-purpose intervention strategies that are likely to be relevant and effective in addressing different types of barriers and enablers. This provides a basis for guiding decision-making during subsequent intervention design and supporting more systematic, targeted and theory-based development of implementation interventions.

In this study, we will therefore use COM-B and the TDF as overarching frameworks to guide our data collection and analysis when exploring influences on current practice and implementation of the E-MOTIVE bundle. Using the COM-B and the TDF, we have mapped potential barriers to E-MOTIVE bundle implementation based on the available literature on bundle implementation (Additional file 1) [17–19], which we plan to iterate throughout the formative research. We will also use the domains from these frameworks to facilitate triangulation and comparison of findings across data sources, participants (i.e. healthcare professional roles), facilities, and countries. We will consult the Behaviour Change Wheel and taxonomy to identify potential implementation strategies to address key barriers and enablers to changing PPH practice and improving implementation of the E-MOTIVE bundle.

### Study objectives

The overall aim of the formative phase of the E-MOTIVE project is to design an implementation strategy for the E-MOTIVE bundle. This study protocol outlines the formative research to support the development of the E-MOTIVE implementation strategy. The specific objectives of the formative phase are:

1. To understand the current clinical management of PPH in the study facilities;
2. To explore PPH detection and management in healthcare providers’ current practice, how detection and treatment may be improved, and potential barriers and enablers to implementation of the E-MOTIVE intervention in their practice; and,
3. To identify and agree with stakeholders the implementation strategies to address identified influences on practices for detecting and managing PPH, and barriers and enablers to uptake of the E-MOTIVE bundle.

## Methods

### Study design and sites

This study protocol outlines the mixed-methods formative phase research activities (Table 1). Given that findings from the formative phase will influence the design and implementation of the E-MOTIVE trial, this study protocol only describes the formative work. There are four methodological components of the formative phase:

1. Qualitative research (in-depth interviews (IDIs);
2. Quantitative survey;
3. Systematic reviews; and
4. Stakeholder consultation and design workshops.

**Table 1:** Formative research methods and data sources

Method	Research question	Data generated	Sites	Participants and sampling	Analysis methods	Forms required
Qualitative research (IDIs)	How is primary PPH during vaginal birth (a) currently detected and managed; and (b) to what extent are the E-MOTIVE bundle components implemented? What are the factors influencing (a) current PPH detection and management; (b) implementation of the E-MOTIVE bundle?	Audio recordings Written transcriptions with field notes	n = 9 facilities total n = 3 per country: Kenya Nigeria South Africa	n = 45 providers total purposively sampled: *Per country (n = 15):* 3 administrators 6 doctors 6 midwives/nurses	Framework analysis using combined deductive framework and inductive thematic analysis	Information sheet Consent form In-depth interview guide
Online Survey	How is primary and refractory PPH during vaginal birth (a) currently detected and managed; and (b) to what extent are the E-MOTIVE bundle components implemented? What are the factors influencing (a) current PPH detection and management; (b) implementation of the E-MOTIVE bundle	Electronic survey data	n = 80 health facilities total Kenya Nigeria South Africa Sri Lanka Tanzania	n = 630–700 healthcare providers total *Per facility (n = 9–10)* 1–2 obstetricians 3 medical officers/ residents/junior doctors 5 midwives/nurses	Descriptive statistics Content analysis (open ended questions)	Information sheet Consent form Survey questionnaire
Stakeholder consultation and design workshops	What are the common and unique factors influencing PPH detection and management across (a) data sources [qualitative and survey]; and (b) countries/sites? To what extent is the proposed E-MOTIVE implementation strategy feasible and acceptable to key stakeholders? How might the proposed strategy be adapted to the local context in each country?	Audio recordings Written transcriptions with field notes Quantitative ranking exercise about feasibility and acceptability	Stakeholder consultation and design workshops will be facilitated for each country	E-MOTIVE international research team, E-MOTIVE country teams, hospital representatives (doctors, midwives)	Thematic analysis & descriptive statistics	Information sheet Consent form Ranking scales

The formative study will take place in five countries: Kenya, Nigeria, South Africa, Sri Lanka and Tanzania. Table 2 presents an overview of the country’s burden of PPH. In line with the Medical Research Council Guidance for Process Evaluations [28], we will balance in-depth qualitative data collection in a sub-sample of three countries, with broader quantitative data collection across all five countries.

**Table 2:** Burden of postpartum hemorrhage and current management in the E-MOTIVE study sites

Global estimates indicate that between the years 2000 and 2017 global maternal deaths reduced by 35%, from 451 000 to 295,000 maternal deaths in 2017 [40]. During this 17 year period the global maternal mortality dropped by 2.9% on average each year. The global lifetime risk of maternal mortality in 2017 was estimated at 1 in 190 [40]
*Nigeria* Nigerian women have a 1 in 21 lifetime risk of maternal death, much higher than the global average. In 2017, 23% of global maternal deaths occurred in Nigeria alone, with 67,000 reported maternal deaths. Nigeria had the fourth highest maternal mortality ratio (MMR) globally in 2017, with 917 deaths per 100,000 live births (Uncertainty Interval (UI) 658 to 1320). With an average Annual Reduction Rate (ARR) point estimate of less than 1.6% (UI -0.8 to 3.5) between 2000 and 2017, Nigeria’s annual rate of reduction in maternal deaths per 100,000 live births dropped at a lower rate than the global average during the same time period [40]. Results from a retrospective study of medical deaths in a tertiary institution in Northern Nigeria indicated that PPH accounted for 14.2% of 112 maternal deaths during a five year period [41]. Further secondary analysis research concluded that PPH was a significant contributor to obstetric hemorrhage and severe maternal outcomes in Nigerian hospitals. PPH occurred in 2.2% of births over a 1 year period, and was the most frequent obstetric complication across all facilities [9]
*Tanzania* The United Republic of Tanzania reported approximately 11,000 (UI 8,100 to 14,000) maternal deaths in 2017, this was the fifth highest number of maternal deaths worldwide. Women in Tanzania are estimated to have a 1 in 36 risk of maternal death. Figures indicate that in 2017, Tanzania was estimated to have an MMR of 524 deaths per 100,000 live births (UI 399 to 712), as well as an ARR point estimate of 2.9% (UI 0.9 to 4.4) between 2000 and 2017, in line with the global average [40]. Retrospective research from 34 public hospitals in Tanzania found that of the 1,987 maternal deaths over a ten year period (2006–2015), 34% were due to eclampsia, followed by 24.6% due to obstetric hemorrhage. During this ten year period, the number of maternal deaths increased, with MMR increasing from 40.24 in 2006 to 57.94 per 100, 000 live births in 2015 [42]. Further descriptive retrospective tertiary research between 2003 and 2012 at a single center in Northern Tanzania reported an MMR of 492.1 per 100,000 live deliveries, in line with previously reported WHO mortality estimates. Postpartum hemorrhage was found to be the leading cause of maternal death during the study period, accounting for 19.2% of maternal mortality [43]
*South Africa* Women in South Africa have a lifetime risk of maternal death of 1 in 330; this risk is lower than the estimated global average. In 2017, the number of maternal deaths in South Africa was estimated to be approximately 1,400. During this year South Africa was estimated to have an MMR point of 119 deaths per 100,000 live births (UI 96 to 153), and an ARR point estimate of 1.7% (UI 0.1 to 3), indicating that the annual rate of reduction fell at a lower rate than the global average between the years 2000 and 2017 [40] The most recent Saving Mothers triennial report, by the National Committee for Confidential Enquiry into Maternal Deaths (NCCEMD) in South Africa, gave obstetric hemorrhage as the cause of 624 or 16.9% of the total deaths between the years 2014 and 2017, making obstetric hemorrhage the third most common cause of maternal death during this period, with 89.5% assessed to have been preventable by better care [44]
*Kenya* Kenyan women have a 1 in 76 risk of maternal death during their lifetime, higher than the global average risk. In the year 2017, it was estimated that 5,000 maternal deaths occurred in Kenya, with an MMR point of 342 deaths per 100,000 live births (UI 253 to 476) during this period. Between the years 2000 and 2017 the WHO estimated that Kenya had an ARR point of 4.3% (UI 2.4 to 5.9), indicating a lower rate of reduction in maternal deaths than the global average during the same time period [40]. In 2017, the Kenyan Ministry of Heath produced their first Saving Mothers Lives report, an enquiry into maternal deaths in Kenyan country and national referral hospitals during the year 2014. Obstetric hemorrhage was found to be the underlying cause of 192, or 40% of the 945 maternal deaths during this period [45]
*Sri Lanka* Sri Lankan women have a lifetime risk of maternal death of 1 in 1,300, and are at lower risk of death than the global average. In 2017, 120 maternal deaths were reported in Sri Lanka. Figures estimate that in 2017, Sri Lanka had an MMR of 36 deaths per 100,000 live births (UI 31 to 41), as well as an ARR point estimate of 2.2% (UI 1.7 to 3.5) between 2000 and 2017 [40]. This indicates that the annual rate of reduction in Sri Lanka dropped at a lower rate than the global average over this 17-year period. Data from the most recent National Maternal Mortality Review from the Family Health Bureau, Ministry of Health Care and Nutrition of Sri Lanka reported that, in 2016 obstetric hemorrhage was the cause of 13.4% of maternal deaths in Sri Lanka, and was the leading cause of maternal death during this period. More specifically, PPH was reported as the cause of death in 8 out of 112 (7.1%) maternal deaths reported in Sri Lanka during this year (46)

## Qualitative research methods

### Study sites and participants

This qualitative IDI study will be conducted in a purposive sample of three health facilities per country across three countries (Kenya, Nigeria, South Africa), for a total of nine health facilities. These health facilities have all met the inclusion criteria for the E-MOTIVE trial, and thus are representative of the types of health facilities participating in the trial. The health facilities included as formative research sites will be excluded from participation in the clinical trial, as they may have been primed or biased as a result of taking part in the formative research. The health facilities to be included as sites in the qualitative study will be selected using maximum variation sampling to ensure variation in the size of the facility, location, representativeness of facility characteristics eligible for main trial, and other key variables.

Healthcare providers currently working on the maternity wards will be included as participants. This includes midwives, nurses, junior doctors, non-physician clinicians, medical officers, residents or trainees, and obstetricians. Healthcare administrators and managers in charge of the maternity wards or health facilities will be included as participants. This may include the head of obstetrics, matron-in-charge, or medical or clinical director. All participants will be capable of responding to the interview questions in English.

### Participant sampling and recruitment

In line with qualitative sample size guidelines based on principles of thematic data saturation [29], we will aim to recruit an initial sample of ten to fifteen participants per country across the three countries (n = a total of 45 participants: n = 15 participants per country, n = 5 participants per health facility). We will analyze data in parallel with data collection, monitoring for thematic data saturation as we go, and adjust the sample size as necessary (e.g. discontinuing further interviews if saturation is deemed achieved, or conducting additional interviews as needed until saturation is deemed achieved). Maximum variation sampling will be used to achieve a stratified sample without random selection and to ensure heterogeneity of research participants. This method uses pre-specified variables to stratify the sample and encourages the recruitment and sampling based on diversity. In each of the selected health facilities, healthcare providers will be sampled based on their cadre, such as nurse, midwife or doctor. The country investigators and research teams will facilitate contact with potentially eligible healthcare providers and administrators at their place of work in the study health facilities. Each individual will be provided with an information sheet about the study, invited to participate by the research team, and if they agree, asked to provide consent.

### Study instruments

We have designed interview topic guides and surveys based on COM-B and the TDF to explore factors influencing current detection and management of PPH, by ensuring we have at least one question per domain of the frameworks. The study instrument will be a semi-structured discussion guide (Additional file 2). This will be structured according to three broad sections:

1. How PPH is currently detected and managed for vaginal birth;
2. Factors influencing current practice for PPH management. These questions will be structured around the domains of the COM-B model (capability, opportunity, motivation) [13]; and
3. Factors potentially influencing the implementation of the E-MOTIVE bundle, structured around the domains of the COM-B model.

The discussion guide has been developed by a team of social and behavioral scientists, obstetricians, and midwives to ensure clinical relevance and use of behavior change theory. The IDIs will be piloted prior to data collection, as part of training the research teams, and revisions made to improve clarity.

### Study procedures and follow-up

All IDIs will be conducted face-to-face, or via Zoom or telephone (currently, COVID-19 restrictions do not allow for face-to-face data collection outside of data collectors’ home institution), in English, and take place in a private setting in the health facility where social distancing and hygienic behaviors can be maintained (or at home if via Zoom or telephone), which is typical and appropriate in each of the study settings. All interviews will be digitally recorded on an encrypted device, and the interviewer will take handwritten field notes containing both descriptive information (settings, actions, behaviors) and reflective information (thoughts, ideas, questions, concerns) about the interview. At the start of the IDIs, participants will be asked to confirm that they have received the information sheet and signed the consent form. IDIs are expected to last approximately 45–60 min and will be conducted by a trained research midwife or local social science researcher.

Once the IDIs are conducted, the study participants will not be followed up. The exception is if the participants in the qualitative study also participate in either the quantitative survey or stakeholder consultation and design workshops (described below). However, participation in the qualitative study will not be contingent on participating in any subsequent research activities, and responses will not be linked by participant or any identifying information.

### Data management and quality assurance

Prior to data collection, a multi-day training session will be conducted for all the research teams, including country principal investigators, data collectors, and other research team members. The training session will include the study objectives, data collection procedures, practice sessions with the tools, and ethical considerations. The international research team and the principal investigators in each country, will train the research teams. This training will take place via Zoom due to COVID-19 travel restrictions. During the data collection period, the country principal investigators will be in consistent communication with the interviewers in the field to respond to any issues that arise during data collection.

All digital recording will be transcribed verbatim into English using a structured format. Transcription will be done by either the data collector or a professional transcription service that is General Data Protection Regulation (GDPR) compliant. Verbatim transcription will be performed close to the time of the completion of the interviews to maintain the uniqueness of the interview without loss of themes. Observations and assessment during the interviews will be written up as field notes and integrated into the transcripts. At the point of transcription, data will be anonymized; no identifiable information about the participants will be included in the written transcripts. Participants and sites will be given identifying numbers for reference. Transcription will occur in parallel to data collection and will be shared on an on-going basis with the international research team to ensure the quality of the data and to determine if certain themes need to be further explored. The international research team will be responsible for reviewing transcripts as they are shared in order to provide ongoing feedback on topics that could be probed more deeply during future interviews, identification of areas for improvement, problem-solving, and facilitating dialogue with the country teams regarding saturation of themes.

### Data analysis plan

Anonymized transcripts will be analyzed using a combined inductive thematic and deductive framework analysis [30]. We plan to first use an inductive thematic analysis approach [31] to allow themes to emerge naturally from the data. Then we will use a deductive framework based on the study objectives and topic guide to map the generated themes to key areas related to PPH detection and management. Lastly, we plan to code the emergent themes representing factors affecting implementation (barriers, facilitators, neutral responses) to the domains of the underlying theoretical framework (COM-B). We will deductively code the generated themes to the relevant COM-B domain that they are judged to best represent. For example, an instance of ‘lack of clear communication and teamwork’ would be mapped to ‘social Opportunity,’ ‘fear and stress managing PPH’ would be mapped to ‘automatic Motivation’, and ‘lack of ready access to oxytocin’ would be mapped to ‘physical Opportunity’. We expect that these themes will represent key influences or factors affecting PPH detection and management, and the implementation of the E-MOTIVE trial and design.

## Quantitative survey design

### Study sites and participants

This will be a cross-sectional, electronic survey conducted in all E-MOTIVE study hospitals (n = 80) across five countries: Kenya, Nigeria, South Africa, Sri Lanka, and Tanzania. These sites have been selected for participation in the E-MOTIVE trial, and will participate in the survey, prior to the trial’s inception. We plan to engage a variety of participants with the survey to gather a wide range of opinions. All potential participants will currently work within the maternity wards of the study facilities, including (but not limited to): midwives, nurses, junior doctors, medical officers, residents, obstetricians, and health managers. Potential participants must be capable of reading and responding to the survey questions in English; there are no restrictions on other demographic characteristics of participants, including: age, gender, race, ethnicity, or sexual orientation. Individuals who are unable or unwilling to give informed consent to participate will not be able to take part, and patients and family caregivers are not eligible to participate in this survey.

### Participant recruitment and sampling

The country principal investigators and facility research coordinators will facilitate contact with the health care providers and managers in each study facility. At each health facility, the facility coordinator will obtain a list of potential participants and their email addresses meeting the eligibility criteria at their site. Individualized email links to complete the survey will be sent to each eligible participant, and reminders to complete the survey will be sent weekly for two weeks. Participants will also be invited to take part, if they have not already, at the study sites, during site visits by the country principal investigators. We have chosen this method of recruitment in order to be able to calculate response rates, understand the denominator, and to facilitate monitoring of responses and follow-up. Responses will not be able to be linked back to an individual’s email address or name.

If participants are willing to take part, they will be asked to provide written consent via the online survey platform (SmartSurvey) before they begin. The first page of the electronic survey will be the participant information sheet. The second page of the electronic survey will be the informed consent form, and a tick box to say ‘I consent to all of the above statements’ or ‘I do not consent to all of the above statements’ Participants will be asked to complete the survey independently and in a setting where they feel comfortable to give honest responses without fear of repercussions.

Maximum variation sampling will be used to achieve a stratified sample without random selection, and to ensure heterogeneity of research participants. This method uses pre-specified parameters to stratify the sample and encourages the recruitment and sampling based on diversity. In each of the 80 study facilities, healthcare professionals will be sampled based on their cadre. We expect the type or designation of health care professionals to vary by facility, but at the minimum would include specialists, medical officers, and midwives/nurses. We plan to sample nine to ten participants per study facility, including five midwives or nurses, three medical officers or junior doctors, and two obstetric specialists, for a total of (n = 720–800) across all sites and countries.

### Study instruments

The study instrument will be an online quantitative survey, hosted by SmartSurvey (Additional file 3). The survey has been developed by a team of social and behavioral scientists, obstetricians and midwives to ensure clinical relevance and use of behavior change frameworks. The survey will be piloted prior to data collection, as part of training the research teams. The survey is expected to take the participants approximately fifteen to twenty minutes to complete. The overarching structure and content of the survey mirrors the qualitative interview guide to facilitate triangulation between the two data collection methods and data sources. The survey will cover the following domains:

1. Sociodemographic information (role, years of experience, country and place of employment);
2. How PPH is currently detected and managed for vaginal birth, including clinical vignettes or scenarios;
3. Factors influencing current practice for PPH management. These questions will be structured around the domains of the COM-B model (capability, opportunity, motivation) [13]; and
4. Potential barriers and enablers to implementing the E-MOTIVE bundle, structured around the domains of the COM-B model.

Response options will include a combination of dichotomous (yes/no or true/false), Likert scales, open-ended short answer response, and multi-option format. The survey will be made available in both a web and mobile friendly format, to enable participants to take part on their own devices with ease.

### Study procedures and follow-up

Participants will be invited to take part in an electronic survey study using a SmartSurvey web link. Participants will be asked during the survey if they would like to hear the results of the survey once they have been collated and interpreted, and will be given the option to provide a contact email if they so choose. If they choose to share their contact email, their email address will be stored separately from their survey responses to protect anonymity.

Once the surveys have been completed, study participants may have additional contact with the research team through two potential avenues. First, if the participant chooses to share their contact email, we will share the study results with them. Second, if the participant is still employed at the study site during the stakeholder consultation and design workshops or the subsequent trial, they may be involved in research workshops, developing implementation strategies, etc. However, they will not be identified as participants in the survey and their decision to participate or not in the survey will have no bearing on their future involvement in the trial. When data collection is complete, the data will be analyzed and the results interpreted. These findings will then be used to inform the development of the E-MOTIVE implementation strategy.

### Data management and quality assurance

All data collected through SmartSurvey is registered under the General Data Protection Regulation 2018. Secure Sockets Layer encryption will be added to any survey, which enables an encrypted link between a web server and a browser to be established. This ensures that all data passed between the web servers and the browsers remain private and integral. All data is stored and backed up on United Kingdom-based servers, and will not be accessed nor shared without prior permission.

Country PIs will be in frequent communication with the E-MOTIVE formative research team in order to respond to any issues that arise during data collection. Halfway through data collection in each country, the E-MOTIVE formative study team will review all data collected up to that point to ensure data quality. Completion rates will be monitored, non-respondents will receive a maximum of two reminder emails asking them to complete the survey. Country principal investigators will also follow up with study sites to maximize response to the survey.

### Data analysis plan

Once data collection is complete and results have been exported from SmartSurvey, data will be cleaned and prepared for analysis in Stata (StataCorp. 2019. Stata Statistical Software: Release 16. College Station, TX: StataCorp LLC). Any missing data or responses of ‘not sure or other’ will be coded to allow this data to be excluded from the analysis where appropriate. Quantitative survey data will be summarized using descriptive statistics as appropriate. Cross-tabulation will be used to describe practices for PPH detection and management, and perceptions of the E-MOTIVE intervention for PPH management. Results will be cross-tabulated and filtered to allow comparison of results by demographic subgroup, including job role, facility type or level, and country. This data will help to establish a benchmark or baseline of practices for PPH management and perceptions of the E-MOTIVE intervention and implementation strategy, to allow comparison of practices and perceptions over the course of the E-MOTIVE trial, and post-trial. For the theory-based items exploring factors influencing PPH detection and management, there will be at least two to three items per domain of the COM-B model. To create a sub-scale score for each domain of COM-B, we will calculate an average response score across items corresponding to that domain [32]. Open-ended or short answer questions will be analyzed using quantitative content analysis [33].

## Systematic reviews

In addition to the primary research outlined above, we plan to conduct several systematic reviews during the formative phase to inform the development of the implementation strategy. First, we plan to conduct a Cochrane qualitative evidence synthesis (systematic review of qualitative evidence) to describe and explore the perceptions and experiences of women, community members, lay health workers, and skilled healthcare providers who have experience with PPH, or with preventing, identifying and managing PPH, in both community and health facility settings [34]. This qualitative evidence synthesis will supplement our primary qualitative research by including the perspectives of women and communities, and understanding the complexities of PPH detection and management further upstream (e.g. for women giving birth at home, in the community, or in primary health facilities). The Cochrane review protocol is published elsewhere [34].

## Stakeholder consultation and design workshops

Design of the E-MOTIVE bundle implementation strategy based on the findings of the formative research will involve two stages: (1) triangulation of findings across data sources by the research team, and (2) stakeholder consultation workshops to refine and adapt the strategy to each country’s local context. The methods for each are described below.

### Research data triangulation

We are using multiple methods to collect data on factors influencing PPH detection and management across countries, and from the perspective of different healthcare professionals. It is therefore important to compare and contrast the findings across these data sources and participant groups using standardized triangulation methods [35, 36]. This will involve the E-MOTIVE research team tabulating findings across data sources and looking for areas of agreement, disagreement, and silence [35]. We will compare findings at three levels: (1) across data sources (interviews vs. surveys vs. systematic review); (2) across countries; (3) across healthcare professional roles (e.g. nurses, midwives, doctors). This will help identify areas for further discussion and clarification in the workshops (see Section: Stakeholder consultation and design workshops below).

It will also inform decisions around components of the implementation strategy that can be standardized/shared across countries, versus those that need to be tailored to each country and/or participant group. Findings from the triangulation exercise will highlight key influences on current practice and barriers and enablers to implementation, which represent potential targets for implementation strategies. To generate potential recommendations for strategies, we will consult the aforementioned tools that pair the COM-B and TDF frameworks as part of the Behaviour Change Wheel to identify potential types of strategies that are likely to be relevant and effective in addressing identified influences, and barriers and enablers. We will generate descriptions of potential strategies, to be presented and discussed at stakeholder consultation workshops (see 5.3.2. below). Where possible, we will build upon existing strategies, such as the Jhpiego “Bleeding after Birth” training package for PPH management.

### Stakeholder consultation and design workshops

We will then host country specific stakeholder consultation and design workshops that bring together the E-MOTIVE international research team and local collaborators. We have already identified a preliminary implementation strategy based on the available evidence on barriers and enablers to bundle implementation in the broader literature (summarized and mapped to COM-B in Additional file 1). During the workshops, we will refine the implementation strategy based on formative findings specific to the study country and PPH contexts.

We anticipate that there will be a standardized set of components (Fig. 1). However, how these are delivered and operationalized may be tailored and refined to each country’s setting and specific influences on PPH detection and management. For example, all countries may receive an element of standardized training, with tailoring in terms of how the training is scheduled, which cadre of healthcare workers attend the training, and how the training is delivered. The workshops will be attended by the E-MOTIVE international research team, E-MOTIVE country teams, and hospital representatives (doctors, midwives). Each participant will be given a study information sheet and asked to sign a consent form prior to participating in the stakeholder consultation and design workshops. At present, it is planned that these workshops will be held virtually, using platforms such as Zoom due to COVID-19 restrictions. However, if safe and appropriate, these may be held face-to-face in each country, or using a hybrid model of Zoom and face-to-face.

The workshop will begin with the E-MOTIVE research team presenting a summary of why PPH is a priority issue to address, an overview of the broader EMOTIVE program of research, and the proposed EMOTIVE bundle. We will then briefly summarize the methods for the formative research and the proposed implementation strategies generated in the previous step. Each strategy will be discussed in turn. For each strategy, we will first outline what the proposed strategy entails, the evidence from the broader literature supporting the strategy, and relevant evidence from the formative research—specifically, what identified challenges (i.e. barriers and enablers) the strategy aims to address. We will ask a series of open and closed questions to participating stakeholders to explore how acceptable and feasible the proposed strategy is, as well as how the implementation strategy could best be delivered, tailored, or adapted to their local contexts. We will audio-record discussions for subsequent analysis. Then the final agreed upon implementation strategy will be summarized using the “template for intervention description and replication” (TIDieR) template, a guideline for describing and reporting complex interventions (e.g. TIDieR checklists; [37]).

Data generated from the workshops will include audio-recordings, transcriptions, and field notes of the discussions. Transcripts and field notes will be analyzed using inductive thematic synthesis. These findings will be used to: (a) systematically and transparently report the intervention development process; and (b) to finalize the manual of operations for the trial, intervention materials and protocols, including training.

## Ethical considerations

We have received ethics approvals for this study (see Declarations: Ethics approval and consent to participate for list of approvals). The study will employ broad participation criteria to be as inclusive as possible of all cadres of healthcare providers. Therefore, specific sub-groups of healthcare providers are not disadvantaged through being unable to participate in the study. All potential participants in both the qualitative and survey components will receive information about the study in plain English, conforming to ethical requirements for research involving human subjects. The language will be easy to understand and free of technical jargons. Participants will be given sufficient time to reflect on the information and ask questions. Those who consent to participate in the study will be requested to sign the informed consent form and it will be made clear that they are free to withdraw from the study at any stage without risk of any negative consequences. All participants will be free to refuse to participate or stop participating at any time, confidentially, and without prejudice. There will be no form of deception in this study.

In the qualitative interviews, the data collector (research midwife and/or in-country social scientists) will facilitate the informed consent process and the paper (hard copy) signed consent forms will be maintained. In the online survey, consent will be obtained via the online platform. The contact details of the local investigators including email address or telephone numbers will be made available to the participants in both the qualitative and survey components, should they require further information and assistance.

Study participants will not receive any compensation for their participation. We expect that the qualitative interviews will take place during their shift at work or from home (if by telephone or Zoom), and they may be provided with light refreshment (such as a cold beverage). Survey participants will not receive any remuneration for their time.

## Discussion

This formative study aims to collect information to inform the design and implementation of a cluster randomized controlled trial and process evaluation on PPH early detection and primary response management, which ultimately has the potential to reduce maternal mortality and severe morbidity in some of the countries with the highest PPH burden globally. Understanding current clinical practice of detection and management of postpartum hemorrhage will provide critical information to ensure that the trial is not only feasible in the study settings, but could be successfully implemented. Understanding potential barriers and enablers and involving the facility health care professionals will help tailor and contextualize the Emotive Bundle implementation strategy.

### Expected study outcomes

In this formative research, we aim to develop an implementation strategy for the E-MOTIVE bundle. The formative research will provide deep understanding of current clinical management practices of PPH, how detection and management can be improved, and how implementing the E-MOTIVE bundle can lead to improved practice. Given the complexities of changing behaviors in order to implement new guidelines and care bundles, using the COM-B and TDF as guiding theoretical frameworks will help us consider the broad range of potential influences on implementation and identify the types of implementation strategies that are likely to be most relevant and effective to target the key influences of appropriate PPH detection and management and implementation of the E-MOTIVE bundle. Moreover, by considering potential barriers and enablers to implementation from the start of the E-MOTIVE project, we expect to maximize the likelihood of a successful intervention, including real-world applicability and effectiveness.

### Strengthening international collaborations

The E-MOTIVE Research Group brings together a diverse team of clinicians, multi-disciplinary researchers, and implementation partners from the University of Birmingham, WHO, University of California San Francisco, University College London, University of Melbourne, Jhpiego, Concept Foundation, Ammalife Charity, University of Liverpool, University of Nairobi, University of Cape Town, University of the Witwatersrand, Bayero University Kano, Nigeria, Muhimbili University of Health and Allied Sciences and University of Kelaniya and University of Jaffna. Each partner brings their own unique expertise and perspectives to the E-MOTIVE Research Group, and this expertise is diffused to the other partners.

### Main problems anticipated and proposed solutions

It is possible that healthcare providers and management may not initially support the implementation of the E-MOTIVE bundle in their workplace, and may therefore be reluctant to take part in research as they may feel unable to express their concerns. However, the study team will remind participants that their names will not be linked to any responses and encourage the study participants to uphold the confidentiality among their peers. We will also remind participants that their decision regarding whether or not to take part in the formative research will not be shared with their peers or line managers. Also, management teams (local champions) will be established in each facility to ensure that top management is aware and supportive of the research. The study team will rely on the country partners and facility staff to identify potential participants.

The E-MOTIVE formative study will be implemented during COVID-19 times, meaning that additional precautions will need to be in place for both the research team and participants. Depending on local conditions and current restrictions, IDIs may take place over Zoom or telephone instead of face-to-face. Where possible, the local research team will minimize face-to-face contact with each other and research participants.

### Next steps following formative phase

Following the stakeholder consultation and design workshops, we plan to field-test the feasibility of the provisional E-MOTIVE implementation strategy, bundle, and tools in two health facilities in each of the five countries (ten health facilities total), over up to two adaptive cycles. Each adaptive cycle will last approximately three months. During the adaptive cycle, we will conduct a small-scale mixed-methods process evaluation to explore two key implementation outcomes [38] relevant to feasibility and pilot studies, which need to be optimized before progressing to the full trial: fidelity (i.e. extent to which the intervention is delivered and engaged with as intended) and acceptability. Following the adaptive cycle, we will convene a meeting with the multi-disciplinary research team members and local clinical collaborators to agree which issues identified need to be addressed, and how best to refine or add to the existing implementation materials. If needed, we may progress to a second adaptive cycle to test the adapted implementation strategy, and further discuss and adapt the intervention as needed. The design of the implementation strategy, adaptive cycle methodology, and results of the mini-process evaluation will be described in subsequent publications.

### Applicability and dissemination of results

The results of this formative research will inform the design and implementation of the E-MOTIVE trial and the process evaluation to be conducted in similar settings. The qualitative and survey components will provide the initial interaction with providers similar to those working in the study sites to better understand current practice around detection and management of postpartum hemorrhage. This evidence will then ensure that the trial implementation plan is feasible, appropriate, and applicable to the context.

We describe our systematic approach to formative research, which considers potential barriers to implementation prior to the start of the E-MOTIVE trial. This systematic approach enables us to design strategies to improve implementation during the trial and beyond. During the trial, this means we are maximizing the likelihood of the interventions being delivered with fidelity If healthcare providers do not use the bundle or if the bundle is not implemented as intended, we have little or no chance of testing the impact of the bundle on reducing PPH-associated mortality and complications [39]. Therefore, a key implication of the formative phase is the opportunity to improve the internal validity of the E-MOTIVE randomized clinical trial, noting its complexity (> 300,000 women across 80 health facilities in five countries).

Moreover, and in line with MRC guidance for developing and evaluating complex interventions [20], this formative protocol outlines our strategy of considering implementation from the start of the E-MOTIVE research program. This includes consideration of what it would take to support roll-out and implementation of the E-MOTIVE bundle in practice, rather than first evaluating the bundle in a trial context then thinking about implementation afterwards. Our approach outlined in this protocol therefore aims to maximize scalability and implementation of the E-MOTIVE bundle in the future.

Findings from this formative study will be disseminated to key stakeholders through a variety of outputs including journal articles, presentations, and evidence briefs as appropriate. Follow the E-MOTIVE study on Twitter @EmotiveTrial.

## Supplementary Information

**Additional file 1. **Mapping initial barriers to E-MOTIVE bundle implementation using COM-B.

**Additional file 2. **In-depth interview guide: healthcare providers.

**Additional file 3. **E-MOTIVE formative survey.

## Data Availability

Not applicable.

## Abbreviations

COM-B: Model of behavior referring to capability, opportunity, and motivation
GDPR: General Data Protection Regulation
IDI: In-depth interview
LMIC: Low- and middle-income countries
TIDieR: Template for intervention description and replication
TDF: Theoretical domains framework
TXA: Tranexamic acid
PPH: Postpartum hemorrhage
WHO: World Health Organization
