# Supplementary material for: Formative research to design an implementation strategy for a postpartum hemorrhage initial response treatment bundle (E-MOTIVE): study protocol
Source: Reprod Health. 2021 Jul 14;18:149. doi: 10.1186/s12978-021-01162-3 (PMC8278177; doi:10.1186/s12978-021-01162-3)
Supplement: Supplementary file 1 — Additional file 1. Mapping initial barriers to E-MOTIVE bundle implementation using COM-B. [file 12978_2021_1162_MOESM1_ESM.pdf]

Appendix 1.

Mapping initial barriers to E-MOTIVE bundle implementation using COM-B

We have identified and mapped some potential barriers to E-MOTIVE bundle implementation from the broader literature (17-19) and mapped these to COM-B. We will refine the list of barriers and enablers based on findings of the formative phase. Initial ideas for strategies to address the identified barriers draw from the Jhpiego "Bleeding after Birth" training package, which has global experience implementing clinical maternal, newborn and child health practices, including prevention, detection, and management of PPH. Likewise, we will learn from other clinical practice behavior change programs in LMICs, such as maternal sepsis bundles in low-resource settings (47), and the Alliance for Innovation on Maternal Health Malawi program (48).

Key barriers to **Capability** (knowledge and skills) include limited knowledge and skills about PPH identification and management, lack of qualified staff, lack of self-efficacy, and inappropriate expectations. To increase Capability and address these barriers, the E-MOTIVE implementation strategy will likely include education and training, such as simulation-based team training at each site, with deliberate skills practice and repeated refresher training.

Key barriers to **social Opportunity** relate to lack of teamwork and ineffective communication between health workers, including while managing PPH. Peer-assisted learning also increases social Opportunity, as it is a cooperative teaching and learning strategy in which learners are active, equal partners, and can facilitate skills practice. It also targets known enablers related to teamwork and communication. We will further increase social Opportunity, and address barriers around lack of engagement of staff or management, and fear of reprisals, by introducing local E-MOTIVE champions. These champions will be responsible for advocating for bundle use locally, providing positive, supportive leadership, and modelling of the desired behaviors. This will also help increase **Motivation**.

Physical Opportunity may be limited by heavy workloads and inefficiencies in workflows, which may compromise early identification and provision of timely and quality care for a woman experiencing PPH. We plan to increase physical Opportunity to implement the E-MOTIVE bundle by restructuring the physical environment of the labor ward through the introduction of trolleys containing the equipment and supplies needed to implement the individual E-MOTIVE bundle components. Enabling easy, timely access to necessary equipment and supplies, and ensuring that trolleys maintain adequate stock, addresses barriers related to workflow and lack of resources.

We also plan introduce calibrated blood collection drapes, which have a visible line marking when 500 ml of blood have been lost. This will address barriers around lack of awareness, inaccurate visual estimations of blood loss, and help increase **Motivation** to implement the E-MOTIVE bundle, by facilitating early detection, and serving as a prompt and decision aid. We will also put up graphical posters and job aids in the ward summarizing the E-MOTIVE bundle components.

Given the relatively limited evidence around implementation of care bundles in low-income settings, and the important of understanding context prior to developing implementation strategies, the formative phase of the E-MOTIVE research program seeks to understand context-specific barriers and enablers to the implementation of the E-MOTIVE bundle in each study site.
