# Supplementary material for: Formative research to design an implementation strategy for a postpartum hemorrhage initial response treatment bundle (E-MOTIVE): study protocol
Source: Reprod Health. 2021 Jul 14;18:149. doi: 10.1186/s12978-021-01162-3 (PMC8278177; doi:10.1186/s12978-021-01162-3)
Supplement: Supplementary file 2 — Additional file 2. In-depth interview guide: healthcare providers. [file 12978_2021_1162_MOESM2_ESM.pdf]

In-depth interview guide: healthcare providers

Thank you very much for taking the time to speak to me today. My name is [interviewer name], and I am one of the E-MOTIVE study team members. Before we begin, can I please confirm that you have received a copy of the study information sheet and consent form?

As a reminder, this study aims to explore how postpartum haemorrhage (PPH) is currently detected and managed in facilities such as this. We are interested in hearing your views and experiences about what currently happens in practice, and what factors influence how PPH is detected and managed. There are no right or wrong answers. Everything you say will be treated confidentially and will not be shared with any of your colleagues, or anyone outside of the E-MOTIVE study team. You are free to answer in as much or as little detail as you wish, to skip over any questions you do not wish to answer, and to pause or stop the interview at any time if needed.

This interview will take approximately one hour- depending on how much you have to say. Can I please check you are free at the moment to talk for this amount of time?

I would also like to please record our conversation- so that I can capture your responses accurately, and so that I can listen to you rather than take many notes. Can I confirm you are happy for me to start recording?

Great. Thank you.

Note to interviewer:

a. Please ask all questions in **BOLD**.
b. Wait before asking the *prompt questions (in italics)* until after the participants has responded to the question in bold.
c. Use the *prompt questions* to get more detailed information about **why** these personal views influence the detection and management of PPH, in addition to how PPH is detected or managed, and who does what and when.

During the interview, please keep track of what's said about any of the following interventions mentioned by the participant when managing PPH:

Massage of uterus ☐ ***Use of Oxytocics*** ☐ ***Use of Tranexamic acid*** ☐ ***Giving IV Fluids*** ☐

This information is required for questions about the E-Motive bundle.

Section 1: Background

We would like to start with understanding a bit about your role in your current job and the facility where you work:

1. **What is your current position**? *[Motivation/ Professional Role & Identity]*
    a. **How long have you been a [doctor/midwife/nurse]?**
    b. **How long have you been in this position at this facility?**

2. **Could you <u>briefly</u> tell me a bit about how the maternity ward in your facility is set-up?** *[Opportunity/Environmental Context & Resources]*
   Prompts: Are there separate rooms for different stages of labour, who works where?

3. **Which parts of the maternity ward do you currently work on?** *[Motivation/ Professional Role & Identity]*
   Prompts: Do you work in antenatal clinic, labour, delivery, postnatal?
    a. **Do you currently work in any other ward?** *[Motivation/ Professional Role & Identity]*
    Prompts: if yes, what do you do?

Thank you for sharing. For the remainder of this interview, I would like to focus on discussing the detection and management of PPH in your facility. For the purpose of this research study, we are not interested in PPH prevention. We will be discussing PPH for vaginal birth only (not caesarean section).

4. **Could you tell me a bit about your understanding of what postpartum haemorrhage (PPH) is?** *[Capability/Knowledge]*
   Prompts: How would you define a PPH?

5. **Could you tell me a bit about your understanding of severe PPH?** *[Capability/Knowledge]*
   a. **How much of an issue do you think PPH is among women giving birth for health care providers and for the facility?** *[Motivation/Goals]*
      Prompts around why, explore what is that makes it an issue or not an issue
   b. **How much of a priority is caring for a woman with PPH in maternity?** *[Motivation/Goals]*
      Prompts: Why is that? Is there anything at a higher priority?
   c. **Have you received any additional training about the detection and management of PPH? In addition to that which you may have received back in [medical/nursing/midwifery] school?** *[Capability/Skills]*
      Prompts: When was the training? Where was the training? (in facility or external); what did the training cover; how was it delivered? (in classroom, online), Did you find it helpful/not helpful?
   d. **Do you feel any additional training is needed on PPH? If so, what would you like to receive training on?** *[Capability/Skills]*
   e. **Do you use any clinical guidelines or protocols for detecting and managing PPH in your facility? If so, which ones?** *[Capability/Memory, Attention & Decision Processes]*
      Prompt: local or national guidelines?
      If guidelines are used: Are they displayed? If not displayed, why and where are they physically located? Is it easy/not easy to access them? How often or when would you use these guidelines/protocols? How useful are guidelines your clinical practice? Why is that?
      If guidelines are not used? Why not?
   f. **Do you feel more guidelines and protocols for detecting and managing PPH are needed?** *[Capability/Behaviour Regulation]*
      Prompts: Why is that? What types of guidelines or protocols are needed?

Section 2. PPH Detection – Vaginal birth

Now I would like to ask you some questions about the detection of postpartum haemorrhage, which is the focus of this research study. Here we are focused only on vaginal birth.

6. **Could you describe how is PPH typically detected in this facility?** *[Capability/Skills]*
   Prompts: What do you do? Who else is involved? What do they do? Where (in which rooms) is PPH usually detected? (e.g. Labour ward, delivery ward, recovery ward, post-natal ward)
7. **Overall, is there anything that makes it difficult for you and your colleagues to detect a PPH? What gets in the way sometimes?** *[Capability/Beliefs about Capabilities]*
   Prompts: Is there anything that helps or makes it easier?
8. **What factors make you suspect that a woman is having PPH?** *[Capability/Knowledge]*
   a. **How do you estimate blood loss?** *[Capability/Skills]*
      Prompt: Why do you use this method? What are the benefits and downsides of this method?
   b. **Can you describe an example of when it was challenging to detect PPH (in this current facility or elsewhere)?** *[Capability/Beliefs about Capabilities]*
   Prompt: Find out why and what made it challenging? What would make it easier to detect?

Section 3. PPH Management – Vaginal birth

Thank you for sharing your views on PPH detection. Now I would like to know a little more about how you manage PPH in your current facility.

9. **Think back to the last time that a woman under your care, with a vaginal birth, had a PPH. Could you describe what happened, what did you do and why did you do it?** *[Capability/Memory, Attention & Decision Processes]*
   Prompts: How long did it take to initiate treatment? How did you make the decision to initiate treatment? Did you work alone or with a team? What did you and your colleagues do to manage the PPH? What tasks did you specifically do?
   [If more than one step of managing PPH]: In what order did you perform these tasks?
   a. **In your opinion, did you work well as a team? Why or why not?** *[Opportunity/Social Influences]*
      Prompts: In general, what gets in the way of team working? What would help you work better as a team when managing a PPH?
10. **Did you lack any resources needed to manage this PPH?** *[Opportunity/Environmental Resources & Context]*
    Prompt: time, staffing, equipment, supplies? Was everything easily available when you needed it? What happens if what you need is not there or is not working?
11. **Did you encounter any challenges or problems when managing this PPH?** *[Capability/Beliefs about Capabilities]*
    Prompts: What could be done to better support you and your colleagues to manage a PPH in this facility?
12. **(If not covered in response to Q7) What would you and your colleagues do if a woman did not respond to initial treatment and she continued to bleed?** *[Capability/Memory, Attention & Decision Processes]*
13. **What are some of the challenges or problems when managing a woman with refractory PPH (haemorrhage that doesn't respond to primary measures)?** *[Capability/Beliefs about Capabilities]*
14. **What are some of the challenges or problems when managing a woman who needs to be moved to operating theatre?** *[Capability/Beliefs about Capabilities; Opportunity/Environmental Context & Resources]*
15. **Do you ever transfer a woman with PPH from this facility to another? If so - What are some of the challenges or problems to transferring a woman to another facility?** *[Capability/Beliefs about Capabilities; Opportunity/Environmental Context & Resources]*
16. **Does this facility ever receive transfers/referrals from lower level facilities? If so, what are some of the challenges or problems you face when receiving a woman transferred/referred from another facility?** *[Capability/Beliefs about Capabilities; Opportunity/Environmental Context & Resources]*

Section 4. The E-MOTIVE bundle

17. **Have you ever heard of a clinical bundle?** *[Capability/Knowledge]*
    If yes, what have you heard?
    If not, define as - A bundle is a set of clinical intervention treatments ALL to be used TOGETHER for every patient with a specific diagnosis.

I would like to ask you some questions about a new specific approach to detect and manage PPH we call the E-MOTIVE Bundle.

Show the E-Motive diagram (copy below) to the participant if F2F or ask them to look at the diagram if using Zoom to be either shared on PC screen or on WhatsApp if using a mobile phone.

READ to the participant all the information about the bundle below. Clearly state the bundle is to be performed ALL AT ONCE not sequentially.

The E-MOTIVE bundle involves performing six evidence-based strategies and interventions to detect and manage PPH, these include:
E *– Early detection of PPH, using a closed end, measuring drape. This drape is calibrated, it has visible line marking when 500 ml of blood has been lost. Once a PPH is detected using the calibrated drape, the following interventions should be performed* ***SIMULTANEOUSLY****:*

M *– Massage of Uterus,*
O *– administration of Oxytocic drug,*
T *– administration of Tranexamic acid,*
IV *– providing IV Fluids and*
E *– Examination of the genital tract + escalation (as necessary).*

These should be performed ***SIMULTANEOUSLY,*** *without waiting for response to individual interventions. If women do not respond to the whole MOTIVE bundle, then progress to treatment for Refractory PPH. You already mentioned that you are already using some or all of these components individually in your facility to manage PPH, but we are interested in discussing how you would use all of these together, at the same time, in a bundle.*

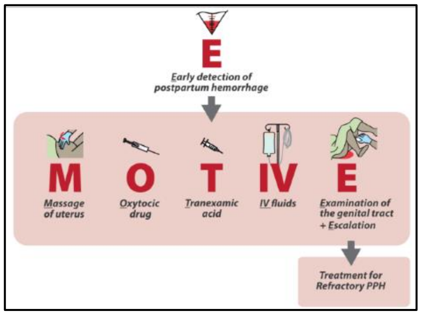

We have already discussed how you detect PPH, here is an example of a calibrated drape.

Show picture of the calibrated drape to the participant (In the same way as described for the E-MOTIVE bundle diagram above)

This is a tool used to help measure blood loss to detect PPH. This drape is calibrated has visible line marking when 500 ml of blood has been lost.

18. **How helpful do you think this would be for detecting of PPH by either yourself or team members?** *[Motivation/Beliefs about Consequences]*
    Prompt: Why or why not? How does this compare to how PPH is currently detected in this facility?
19. **What would be needed to help introduce this calibrated drape in your facility?** *[Capability/Behaviour Regulation]*
    Prompts: Would there be any challenges that you could anticipate?

After PPH is detected, the treatments in the bundle are massage of uterus, use of oxytocics and tranexamic acid and giving IV fluids all delivered ***all at once****.*

20. **Can I please confirm which of these you currently use in this facility?** *[Capability/Memory, Attention & Decision Processes]*
    a. **Do you use some treatments in the bundle more often than others? Why is that?**
21. **Does your team currently use any of these treatments all at once?**
    If yes, ask:
    a. **Did you and your team have to make any changes in order to do these treatments all at once? What are the challenges to using the treatments all at once?** *[Capability/Behaviour Regulation]*

Prompts: What were these changes? Which treatments are used in combination or not?

22. **How confident do you feel about performing all these treatments in the bundle at once?** *[Capability/Beliefs about Capabilities]*
23. **What would make you more or less confident?** *[Capability/Beliefs about Capabilities]*
    a. **Are there any treatments you feel more or less confident doing?**
24. **In your opinion, are any of the treatments in the bundle more or less important than the others? Why is that?** *[Motivation/Goals]*
25. **Which of the treatments in the bundle are not currently used?** *[Capability/Memory, Attention & Decision Processes]*

*For **each** treatment not used (i.e. massage of uterus, use of oxytocins, use of tranexamic acid & giving IV fluids) mentioned ask:*

26. **Do you think there would be any challenges or issues to using [treatment]? What would those be?** *[Capability/Beliefs about Capabilities]*
27. **If you and your colleagues were asked to use these treatments all together when managing PPH, as described in the bundle, what would you need to help you do it?** *[Capability/ Beliefs about Capabilities]*
    a. **To what extent would you know what to do?** *[Capability/Knowledge]*
    b. **To what extent would you need additional skills?** *[Capabilities/Skills]*
    c. **Would you have everything you need to perform the bundle?** *[Opportunity/Environmental Context & Resources]*
    d. **What do you think are the potential benefits of managing PPH using such a bundle?** *[Motivation/Beliefs about Consequences]*
    e. **What do you think are the potential disadvantages of managing PPH using such a bundle?** *[Motivation/Beliefs about Consequences]*
    f. **To what extent do you think managing PPH as described in the bundle, is likely to help manage PPH more effectively?** *[Motivation/Beliefs about Consequences]*
    g. **How would managing PPH as described in the bundle affect the ways you work as a team?** *[Motivation/Beliefs about Consequences]*

Moving on the final part of the bundle

28. **Does your team currently examine the woman's genital tract as part of PPH management?** *[Capability/Memory, Attention & Decision Processes]*
    If yes, ask:
    a. **Who examines the woman's genital tract?** *[Motivation/Professional Role & Identity]*
    b. **How and when do you do the examination?** *[Capability/Memory, Attention & Decision Processes]*
    c. **Are there any challenges to performing this examination? Does anything ever get in the way of you doing these examinations?** *[Capability/Beliefs about Capabilities]*
    If no, ask:
    a. **Why is it not done? Is there anything that makes it difficult to do?** *[Capability/Beliefs about Capabilities]*
    b. **What would help you to do this in practice?** *[Capability/Beliefs about Capabilities]*

Section 5. Conclusion

Thank you for your responses. We are now moving on to the final section of the interview where I have a few more general questions about PPH.

29. **Does your team get any feedback on how you have detected and managed PPH?** *[Capability/Behaviour Regulation]*
    If yes, ask:
    a. **From whom?** *[Motivation/Professional Role & Identity]*

b. **What does the feedback cover?** *[Capability/Knowledge]*
c. **How useful do you feel this feedback is?** *[Motivation/Beliefs about Consequences]*
d. **What could be done to make this feedback more useful?** *[Motivation/Beliefs about Consequences]*

If no, ask:

a. **Do you think having feedback would be useful?** *[Motivation/Beliefs about Consequences]*

30. **How do you feel when you are managing a PPH? Why is that?** *[Motivation/Emotion]*
    Prompts: To what extent is managing PPH something you find stressful? Why is that?
31. **How do you feel when something goes wrong or there is a complication?** *[Motivation/Emotion]*
    Prompt: Why is that? Is there anything that could be done to help or support you and your colleagues in this situation?
32. **Have you ever been concerned about complaints from the community about the care they received?** *[Motivation/Emotion]*
    Prompts: What were the complaints? Why do you think they complained? How did you feel about the complaints if you were personally involved?
33. **Do you know or have you ever heard of someone being disciplined for inadequate detection and/or management of PPH?** *[Motivation/Reinforcement]*
34. **To what extent does disciplinary action being taken on staff or on the hospital influence the detection and management of PPH in this facility?** *[Motivation/Reinforcement]*
    a. **How does this impact on what you have told me about how staff do your job?**
35. **In your opinion, what do you think would happen if PPH was not detected and managed in your facility?** *[Motivation/Beliefs about Consequences]*
    Prompts: Would there be any consequences? For whom?
36. **Are you aware of any strategies in your facility to try to improve detection and management of PPH?** *[Capability/Behaviour Regulation]*
    a. **If yes, please explain and in your opinion, how effective have these strategies been?**
37. **Lastly, I would just like to quickly ask to what extent has the COVID-19 outbreak impacted on maternal care in your facility?**
    Prompts: Do fewer women come to the facility? Have maternity staff been seconded to other wards? Have you cared for women with Covid-19? Do you have to wear personal protection equipment? Have you received training on how to cope with Covid-19 in general maternity wards and for managing PPH?
38. **Finally, is there anything else that you would like to share with me about anything we discussed today?**

**Field notes:** Please remember to write down some brief field notes at the end of the interview. This provides helpful contextual information about the interview, that will help with analysis. Thank you.
As you are conducting IDIs during a pandemic time, please, provide a bit of context:<br>On **procedures of contacting the respondents** (any challenges/ difficulties you faced? How did you overcome those? Did you have to contact the eligible respondent multiple times? Etc.)
**Interaction between interviewer and interviewee: How did you feel about the interactions between you and the respondent**? (i.e. was s/he spontaneous? Did you feel the respondent was shy/uncomfortable in providing detail on any Qs? Could you recognize any reasons of her/him being uncomfortable? Please, list all the possible reasons that you identified.<br>How did you manage those situations and encourage the respondent to respond? Do you think your professional background (being a health professional) had an influence on the respondent?)
**Researcher reflection and identification of bias:** Please, spend a few moments reflecting on the whole of the interview and your performance as a participant in the narrative and as an interviewer. **What went well/not well and why? Do you think you missed any important Qs/prompts?** If so, please note down immediately and make sure you ask those in your next interview.
