# Supplementary material for: Formative research to design an implementation strategy for a postpartum hemorrhage initial response treatment bundle (E-MOTIVE): study protocol
Source: Reprod Health. 2021 Jul 14;18:149. doi: 10.1186/s12978-021-01162-3 (PMC8278177; doi:10.1186/s12978-021-01162-3)
Supplement: Supplementary file 3 — Additional file 3. E-MOTIVE formative survey. [file 12978_2021_1162_MOESM3_ESM.pdf]

E-MOTIVE Formative Survey

Country Selection

Please select the country you are based in: *

- ☐ Kenya
- ☐ Nigeria
- ☐ South Africa – Eastern Cape
- ☐ South Africa – Western Cape
- ☐ South Africa – KwaZulu-Natal
- ☐ Sri Lanka
- ☐ Tanzania

Local Contact Details (Kenya)

The E-MOTIVE Principal Investigator in Kenya is [redacted]
The E-MOTIVE study co-ordinator in Kenya is [redacted]

If you have any problems and need help completing this survey please contact one of the above.

What is the name of the facility you currently work at? *

Local Contact Details (Nigeria)

The E-MOTIVE Principal Investigator in Nigeria is [redacted]
The E-MOTIVE Trial Coordinator in Nigeria is [redacted]

If you have any problems and need help completing this survey please contact one of the above.

What is the name of the facility you currently work at? *

Local Contact Details (South Africa - Eastern Cape)

The E-MOTIVE Principal Investigator in South Africa is [redacted]
The E-MOTIVE Trial Coordinator in South Africa (Eastern Cape) is [redacted]

If you have any problems and need help completing this survey please contact one of the above.

What is the name of the facility you currently work at?

Local Contact Details (South Africa - Western Cape)

The E-MOTIVE Principal Investigator in South Africa is [redacted]
The E-MOTIVE Co-Principal Investigator in South Africa (Western Cape) is [redacted]

The E-MOTIVE Trial Coordinator in South Africa (Western Cape) is [redacted]

If you have any problems and need help completing this survey please contact one of the above.

What is the name of the facility you currently work at?

Local Contact Details (South Africa - KwaZulu-Natal)

The E-MOTIVE Principal Investigator in South Africa is [redacted]
The E-MOTIVE Trial Coordinators in South Africa (KwaZulu-Natal) are [redacted] and [redacted]

If you have any problems and need help completing this survey please contact one of the above.

What is the name of the facility you currently work at? *

Local Contact Details (Sri Lanka)

The E-MOTIVE Principal Investigator in Sri Lanka is [redacted]

The E-MOTIVE Trial Coordinator in Sri Lanka is [redacted]

If you have any problems and need help completing this survey please contact one of the above.

What is the name of the facility you currently work at? *

Local Contact Details (Tanzania)

The E-MOTIVE Principal Investigator in Tanzania is [redacted]

The E-MOTIVE Trial Coordinators in Tanzania are [redacted] and [redacted]

If you have any problems and need help completing this survey please contact one of the above.

What is the name of the facility you currently work at? *

Demographics

For the purpose of this survey we will be focusing on the <u>**detection and management**</u> of postpartum haemorrhage after vaginal births in your hospital. By detection we are referring to the

initial diagnosis of postpartum haemorrhage. This survey is not focused on the prevention of postpartum haemorrhage.

There are no right or wrong answers to any of the questions asked in this survey, we are simply interested in exploring your personal experience and beliefs surrounding postpartum haemorrhage. We want to reassure you that the answers you give will not be able to be linked back to you, and will remain anonymous and confidential.

We would like to start with a few questions about your role in your current job, and the facility where you work. Please work through the questions on this page before progressing on to the next section of questions.

What is your current position? *

- ☐ Consultant (or specialist doctor)
- ☐ Medical doctor
- ☐ Medical doctor in training - resident
- ☐ Medical doctor in training - house officer
- ☐ Medical student
- ☐ Clinical/medical officer (non-physician clinician)
- ☐ Nurse
- ☐ Nursing student
- ☐ Midwife
- ☐ Midwifery student
- ☐ Nurse-Midwife
- ☐ Nurse-Midwifery student
- ☐ Other (please specify): ____________

How long, in years, have you worked in this position at this hospital? *

____

How long, in years, have you worked in this position in total? Note: The answer you provide must be equal or greater than your answer to the previous question *

____

When did you last perform or assist with a vaginal delivery? *

- ☐ In the last week
- ☐ In the last month
- ☐ In the last 3 months
- ☐ In the last 6 months
- ☐ Over 6 months ago

- [ ] I have never performed or assisted with a vaginal delivery

When was the last time you detected and/or managed a case of postpartum haemorrhage? *

- [ ] In the last week
- [ ] In the last month
- [ ] In the last 3 months
- [ ] In the last 6 months
- [ ] Over 6 months ago
- [ ] I have never detected and/or managed a case of postpartum haemorrhage

Training

Next, we would like to focus on the training you have received around detecting and managing postpartum haemorrhage.

Have you received any training about postpartum haemorrhage detection and/or management? * *[Capability, Physical]*

- [ ] Yes - **detection** of postpartum haemorrhage training only
- [ ] Yes - **management** of postpartum haemorrhage training only
- [ ] Yes - postpartum haemorrhage **detection <u>and</u> management** training
- [ ] No postpartum haemorrhage training
- [ ] Unsure

How long ago did you receive your most recent training? * *[Capability, Physical]*

- [ ] Less than one year ago
- [ ] 1-2 years ago
- [ ] 3-5 years ago
- [ ] Over 5 years ago

Please think back to your most recent postpartum haemorrhage training.

Was your most recent postpartum haemorrhage training given by...? * *[Capability, Physical]*

- [ ] Trainers from your own hospital
- [ ] Trainers from the district or regional team
- [ ] Trainers from another organisation e.g. a Non-Governmental Organisation (NGO)
- [ ] Trainers from the Ministry of Health

☐ I don't know

☐ Other (please specify):

What was the format of the training? Please tick all that apply. * *[Capability, Physical]*

☐ Workshop

☐ Presentation

☐ Simulation

☐ Case based

☐ Lectures

☐ Textbook

☐ Other (please specify):

Training

To what extent do you agree with the following statements...? *

	Strongly disagree	Disagree	Neither agree nor disagree	Agree	Strongly agree	I have not received training
I think that the postpartum haemorrhage training I received was useful *[Capability, Physical]*	☐	☐	☐	☐	☐	☐
I have been adequately trained how to **detect** postpartum haemorrhage after a vaginal birth *[Capability, Physical]*	☐	☐	☐	☐	☐	☐
I have been adequately trained how to **manage** postpartum haemorrhage after a vaginal birth *[Capability, Physical]*	☐	☐	☐	☐	☐	☐

Guidelines

We would now like to ask you a few questions about postpartum haemorrhage guidelines.

To clarify, guidelines are any local, hospital-specific, national, or international recommendations, steps, procedures or protocols for detecting and managing postpartum haemorrhage.

Are there clinical practice guidelines used in your facility to detect and manage postpartum haemorrhage? * *[Capability, Psychological]*

☐ Yes

☐ No

Are these clinical practice guidelines on display (e.g. posters, on a bulletin board)? * *[Opportunity, Physical]*

- [ ] Yes
- [ ] No

Where are the guidelines for postpartum haemorrhage detection and management displayed? Please tick all that apply. * *[Opportunity, Physical]*

- [ ] Labour and/or delivery rooms
- [ ] Tea room / break room
- [ ] Meeting room for clinical staff
- [ ] Other (please specify):

To what extent do you agree or disagree with the following statements...? *

	Strongly disagree	Disagree	Neither agree nor disagree	Agree	Strongly agree
I am aware of recommendations and guidelines for the management and detection of postpartum haemorrhage *[Capability, Psychological]*	☐	☐	☐	☐	☐
I use clinical guidelines when I am detecting and managing a postpartum haemorrhage *[Capability, Psychological]*	☐	☐	☐	☐	☐
I find recommendations and guidelines for detecting and managing postpartum haemorrhage useful for my clinical practice *[Capability, Psychological]*	☐	☐	☐	☐	☐
I can easily access a copy of clinical guidelines for detecting and managing postpartum haemorrhage in my hospital *[Opportunity, Physical]*	☐	☐	☐	☐	☐

Detecting Postpartum Haemorrhage

The next section of the survey is about how postpartum haemorrhage is **detected** after a vaginal birth at your hospital. *By detection we are referring to the initial diagnosis of postpartum haemorrhage.*

Is detecting postpartum haemorrhage after a vaginal birth part of your clinical role? * *[Motivation, Reflective]*

- [ ] Yes
- [ ] No

After a vaginal birth, how is a postpartum haemorrhage typically detected at your hospital? Please tick all that apply. * *[Capability, Psychological]*

- ☐ Visual estimation of blood loss
- ☐ Collecting and measuring blood using a tool
- ☐ Using vital signs, including: blood pressure, pulse, heart rate, respirations
- ☐ Using uterine tone and size
- ☐ CRADLE device
- ☐ Other (please specify): ____________

Which tools would you use to measure blood lost after a vaginal birth? Please tick all that apply. * *[Capability, Psychological, and Opportunity, Physical]*

- ☐ Kidney basin
- ☐ Bed pan
- ☐ Counting of soaked swabs, gauzes, sponges, and/or linen
- ☐ Weighing of soaked swabs, gauzes, sponges, and/or linen
- ☐ Kelly's pad with basin
- ☐ Obstetric drape
- ☐ Cloth mat
- ☐ Khanga/Kanga method
- ☐ Q-mat
- ☐ Other (please specify): ____________

In your opinion, how important are each of these methods in helping you to detect a postpartum haemorrhage? * *[Motivation, Reflective]*

	Not at all important	Slightly important	Moderately important	Very important	Extremely important	I have never used this method
Visual estimation of blood loss	☐	☐	☐	☐	☐	☐
Collecting and measuring blood using a tool	☐	☐	☐	☐	☐	☐
Using vital signs, including blood pressure, pulse, heart rate, respirations	☐	☐	☐	☐	☐	☐
Modified Early Obstetric Warning System (MEOWS)	☐	☐	☐	☐	☐	☐
CRADLE device	☐	☐	☐	☐	☐	☐
Using uterine tone and size	☐	☐	☐	☐	☐	☐

To what extent do you agree or disagree with the following statements...? *

	Strongly disagree	Disagree	Neither agree nor disagree	Agree	Strongly agree
My team is disciplined for failing to detect a PPH during a vaginal birth *[Motivation, Automatic]*	☐	☐	☐	☐	☐
My emotional state affects how I detect a PPH *[Motivation, Automatic]*	☐	☐	☐	☐	☐
When I detect a PPH during a vaginal birth I receive feedback on my performance *[Capability, Psychological]*	☐	☐	☐	☐	☐
I know what I need to do to detect a postpartum haemorrhage after a vaginal birth *[Capability, Psychological]*	☐	☐	☐	☐	☐
I get sufficient feedback about my performance when I detect a PPH during a vaginal birth *[Capability, Psychological]*	☐	☐	☐	☐	☐
I want to improve how I detect PPH during a vaginal birth *[Motivation, Reflective]*	☐	☐	☐	☐	☐
Fear of repercussions from the patient and their family affect how I detect a PPH *[Motivation, Automatic]*	☐	☐	☐	☐	☐
If I need help detecting a postpartum haemorrhage after a vaginal birth, I can easily get the support or assistance I need from my team *[Opportunity, Social]*	☐	☐	☐	☐	☐
I have the skills to detect postpartum haemorrhage after vaginal birth *[Capability, Physical]*	☐	☐	☐	☐	☐
In the past, I have needed assistance when detecting a postpartum haemorrhage after a vaginal birth, as I did not have the competency required *[Motivation, Reflective]*	☐	☐	☐	☐	☐
My emotions are negatively affected by detecting a PPH *[Motivation, Automatic]*	☐	☐	☐	☐	☐
I intend to improve my knowledge of PPH detection during vaginal birth *[Motivation, Reflective]*	☐	☐	☐	☐	☐
I am confident that I can detect a postpartum haemorrhage after a vaginal birth, even when there is little time *[Motivation, Reflective]*	☐	☐	☐	☐	☐

Improving PPH detection is not discussed in regular meetings at my facility *[Capability, Psychological]*	☐	☐	☐	☐	☐
If I successfully detect a PPH after a vaginal birth at my health facility I am commended *[Motivation, Automatic]*	☐	☐	☐	☐	☐
I feel good if I successfully detect a PPH during a vaginal birth *[Motivation, Automatic]*	☐	☐	☐	☐	☐
It is easy to accurately estimate the volume of blood lost after a vaginal birth *[Capability, Psychological]*	☐	☐	☐	☐	☐
It is easy to distinguish between normal blood loss and a postpartum haemorrhage after a vaginal birth *[Capability, Psychological]*	☐	☐	☐	☐	☐

Managing Postpartum Haemorrhage

The next section of this survey is about how postpartum haemorrhage is **managed** after vaginal birth at your facility.

Is managing postpartum haemorrhage after a vaginal birth part of your clinical role? * *[Motivation, Reflective]*

☐ Yes
☐ No

You have detected a postpartum haemorrhage following a vaginal birth. We are now interested in your initial response.

By initial response we mean all actions or interventions that you would always perform after a postpartum haemorrhage all at once or in quick succession before reassessing the woman.

What would be your initial response? Please tick all that apply. * *[Capability, Psychological]*

☐ Admission to ICU
☐ Administer ergometrine
☐ Repair of high vaginal or cervical tears
☐ Uterine massage
☐ Administer tranexamic acid (TXA)
☐ Blood pressure and pulse check
☐ Manual removal of placenta
☐ Administer carboprost

- [ ] Administer IV fluids
- [ ] Non-pneumatic anti-shock garment (NASG)
- [ ] Internal examination in delivery room
- [ ] Bimanual compression
- [ ] Uterine balloon tamponade
- [ ] Administer misoprostol
- [ ] Administer oxytocin
- [ ] Administer carbetocin
- [ ] Internal uterine exploration in theatre
- [ ] Other (please specify):

How important do you think each of these actions or interventions are in helping to manage postpartum haemorrhage after a vaginal birth? ** [Motivation, Reflective]*

	Not at all important	Slightly important	Moderately important	Very important	Extremely important
Admission to ICU	☐	☐	☐	☐	☐
Administer ergometrine	☐	☐	☐	☐	☐
Repair of high vaginal or cervical tears	☐	☐	☐	☐	☐
Uterine massage	☐	☐	☐	☐	☐
Administer tranexamic acid (TXA)	☐	☐	☐	☐	☐
Blood pressure and pulse check	☐	☐	☐	☐	☐
Manual removal of placenta	☐	☐	☐	☐	☐
Administer carboprost	☐	☐	☐	☐	☐
Administer IV fluids	☐	☐	☐	☐	☐
Non-pneumatic anti-shock garment (NASG)	☐	☐	☐	☐	☐
Internal examination in delivery room	☐	☐	☐	☐	☐
Bimanual compression	☐	☐	☐	☐	☐
Uterine balloon tamponade	☐	☐	☐	☐	☐
Administer misoprostol	☐	☐	☐	☐	☐
Administer oxytocin	☐	☐	☐	☐	☐
Administer carbetocin	☐	☐	☐	☐	☐
Internal uterine exploration in theatre	☐	☐	☐	☐	☐

How often do you and/or your team do the following when managing a postpartum haemorrhage following vaginal birth? * *[Capability, Psychological]*

	Never	Rarely	Sometimes	Often	Always
Admission to ICU	☐	☐	☐	☐	☐
Administer ergometrine	☐	☐	☐	☐	☐
Repair of high vaginal or cervical tears	☐	☐	☐	☐	☐
Uterine massage	☐	☐	☐	☐	☐
Administer tranexamic acid (TXA)	☐	☐	☐	☐	☐
Blood pressure and pulse check	☐	☐	☐	☐	☐
Manual removal of placenta	☐	☐	☐	☐	☐
Administer carboprost	☐	☐	☐	☐	☐
Administer IV fluids	☐	☐	☐	☐	☐
Non-pneumatic anti-shock garment (NASG)	☐	☐	☐	☐	☐
Internal examination in delivery room	☐	☐	☐	☐	☐
Bimanual compression	☐	☐	☐	☐	☐
Uterine balloon tamponade	☐	☐	☐	☐	☐
Administer misoprostol	☐	☐	☐	☐	☐
Administer oxytocin	☐	☐	☐	☐	☐
Administer carbetocin	☐	☐	☐	☐	☐
Internal uterine exploration in theatre	☐	☐	☐	☐	☐

To what extent do you agree or disagree with the following statements...? *

	Strongly disagree	Disagree	Neither agree nor disagree	Agree	Strongly agree
If I need help managing a postpartum haemorrhage after a vaginal birth, I can easily get the support or assistance I need from my team *[Opportunity, Social]*	☐	☐	☐	☐	☐
Fear of repercussions from the patient and their family affect how I manage a PPH *[Motivation, Automatic]*	☐	☐	☐	☐	☐

	Strongly disagree	Disagree	Neither agree nor disagree	Agree	Strongly agree
I get sufficient feedback about my performance when I manage a PPH *[Capability, Psychological]*	☐	☐	☐	☐	☐
My team is disciplined for failing to manage a PPH during a vaginal birth *[Motivation, Automatic]*	☐	☐	☐	☐	☐
My emotions are negatively affected by managing a PPH *[Motivation, Automatic]*	☐	☐	☐	☐	☐
I am confident that I can manage a postpartum haemorrhage after a vaginal birth, even when there is little time *[Motivation, Reflective]*	☐	☐	☐	☐	☐
I have the skills to manage a postpartum haemorrhage after a vaginal birth *[Capability, Physical]*	☐	☐	☐	☐	☐
I want to improve how I manage PPH during a vaginal birth *[Motivation, Reflective]*	☐	☐	☐	☐	☐
If I successfully manage a PPH at my health facility I am commended *[Motivation, Automatic]*	☐	☐	☐	☐	☐
I feel good if I successfully manage a PPH during a vaginal birth *[Motivation, Automatic]*	☐	☐	☐	☐	☐
Improving PPH management is not discussed in regular meetings at my facility *[Capability, Psychological]*	☐	☐	☐	☐	☐
I intend to improve my knowledge of PPH management *[Motivation, Reflective]*	☐	☐	☐	☐	☐
My emotional state affects how I manage a PPH *[Motivation, Automatic]*	☐	☐	☐	☐	☐
In the past, I have needed assistance when managing a postpartum haemorrhage after a vaginal birth, as I did not have the competency required *[Motivation, Reflective]*	☐	☐	☐	☐	☐
I know what I need to do to manage a postpartum haemorrhage after a vaginal birth *[Capability, Psychological]*	☐	☐	☐	☐	☐
When I manage a PPH I receive feedback on my performance *[Capability, Psychological]*	☐	☐	☐	☐	☐

Refractory Postpartum Haemorrhage

We are now interested in finding out what you would do if the initial response is unsuccessful in managing the postpartum haemorrhage, and the woman continues to bleed. We are calling this your 'next response'.

To clarify, by next response we mean all actions or interventions that you would always perform as a second-line response if the woman continues to bleed despite the first response. If you ticked any of these options as your first response, please only tick here if you would do this again in your second response e.g. if you would administer a second dose of oxytocin.

What would be your next response? Please tick all that apply. * *[Motivation, Reflective]*

- ☐ Admission to ICU
- ☐ Administer ergometrine
- ☐ Repair of high vaginal or cervical tears
- ☐ Uterine massage
- ☐ Administer tranexamic acid (TXA)
- ☐ Blood pressure and pulse check
- ☐ Manual removal of placenta
- ☐ Administer carboprost
- ☐ Administer IV fluids
- ☐ Non-pneumatic anti-shock garment (NASG)
- ☐ Internal examination in delivery room
- ☐ Bimanual compression
- ☐ Uterine balloon tamponade
- ☐ Administer misoprostol
- ☐ Administer oxytocin
- ☐ Administer carbetocin
- ☐ Internal uterine exploration in theatre
- ☐ Other (please specify):

Detecting and Managing Postpartum Haemorrhage

To what extent do you agree or disagree with the following statements *

	Strongly disagree	Disagree	Neither agree nor disagree	Agree	Strongly agree	Unsure
Postpartum haemorrhage is a problem for women giving birth vaginally in my hospital *[Motivation, Reflective]*	☐	☐	☐	☐	☐	☐
Postpartum haemorrhage is something that concerns me *[Motivation, Automatic]*	☐	☐	☐	☐	☐	☐
I find detecting and managing postpartum haemorrhage stressful *[Motivation, Automatic]*	☐	☐	☐	☐	☐	☐
Postpartum haemorrhage is a priority compared to my other clinical responsibilities in my current role *[Motivation, Reflective]*	☐	☐	☐	☐	☐	☐

Availability

The next section will ask how readily available different tools and treatments are in your hospital.

Measuring Tool

At your hospital, how readily available are...? * *[Opportunity, Physical]*

	Never available	Rarely available	Sometimes available	Often available	Always available
Tools to measure the volume of blood lost	☐	☐	☐	☐	☐

Why are tools to measure the volume of blood lost not readily available? Please tick all that apply. * *[Opportunity, Physical, and Capability, Physical]*

☐ Not routinely stocked

☐ Inconsistent supplies

☐ Incorrect supplies

☐ Not easily located when needed

☐ Storage issues - lack of space

☐ Inadequate staffing

☐ Other (please specify): ____________

Uterotonic Drugs

At your hospital, how readily available are...? *[Opportunity, Physical]*

	Never available	Rarely available	Sometimes available	Often available	Always available
Uterotonic drugs e.g. oxytocin	☐	☐	☐	☐	☐

Why are uterotonic drugs not readily available? Please tick all that apply. * *[Opportunity, Physical, and Capability, Physical]*

- ☐ Not routinely stocked
- ☐ Inconsistent supplies
- ☐ Incorrect supplies
- ☐ Not easily located when needed
- ☐ Storage issues - no refrigeration
- ☐ Storage issues - lack of space
- ☐ No trained staff to administer tranexamic acid
- ☐ Other (please specify): ____________

Tranexamic Acid (TXA)

At your hospital, how readily available is...? * *[Opportunity, Physical]*

	Never available	Rarely available	Sometimes available	Often available	Always available
Tranexamic acid (TXA)	☐	☐	☐	☐	☐

Why is tranexamic acid (TXA) not readily available? Please tick all that apply. *
[Opportunity, Physical, and Capability, Physical]

- ☐ Not routinely stocked
- ☐ Inconsistent supplies
- ☐ Incorrect supplies
- ☐ Not easily located when needed
- ☐ Storage issues - lack of space
- ☐ No trained staff to administer tranexamic acid
- ☐ Other (please specify): ____________

IV Fluids

At your hospital, how readily available are...? * *[Opportunity, Physical]*

	Never available	Rarely available	Sometimes available	Often available	Always available
IV fluids	☐	☐	☐	☐	☐

Why are IV fluids not readily available? Please tick all that apply. * *[Opportunity, Physical, and Capability, Physical]*

- ☐ Not routinely stocked
- ☐ Inconsistent supplies
- ☐ Incorrect supplies
- ☐ Not easily located when needed
- ☐ Storage issues - lack of space
- ☐ No trained staff to administer IV fluids
- ☐ Other (please specify):

Blood Transfusion

At your hospital, how readily available are...? * *[Opportunity, Physical]*

	Never available	Rarely available	Sometimes available	Often available	Always available
Blood transfusions	☐	☐	☐	☐	☐

Why are blood transfusions not readily available? Please tick all that apply. * *[Opportunity, Physical, and Capability, Physical]*

- ☐ Not routinely stocked
- ☐ Inconsistent supplies
- ☐ Incorrect supplies
- ☐ Time constraints with preparing blood
- ☐ Storage issues - lack of space
- ☐ No trained staff to administer blood transfusion
- ☐ Other (please specify):

Non-Pneumatic Anti-Shock Garments (NASG)

At your hospital, how readily available are...? * *[Opportunity, Physical]*

	Never available	Rarely available	Sometimes available	Often available	Always available
Non-pneumatic anti-shock garments (NASG)	☐	☐	☐	☐	☐

Why are non-pneumatic anti-shock garments (NASG) not readily available? Please tick all that apply. *[Opportunity, Physical, and Capability, Physical]*

☐ Not routinely stocked

☐ Inconsistent supplies

☐ Incorrect supplies

☐ Not easily located when needed

☐ Storage issues - lack of space

☐ No trained staff to apply NASG

☐ Other (please specify):

Uterine Balloon Tamponades (UBT)

At your hospital, how readily available are...? * *[Opportunity, Physical]*

	Never available	Rarely available	Sometimes available	Often available	Always available
Uterine balloon tamponades (UBT)	☐	☐	☐	☐	☐

Why are uterine balloon tamponades (UBT) not readily available? Please tick all that apply. *[Opportunity, Physical, and Capability, Physical]*

☐ Not routinely stocked

☐ Inconsistent supplies

☐ Incorrect supplies

☐ Not easily located when needed

☐ Storage issues - lack of space

☐ No trained staff to insert the uterine balloon tamponade

☐ Other (please specify):

Laparotomy

At your hospital, how readily available is...? *[Opportunity, Physical]

	Never available	Rarely available	Sometimes available	Often available	Always available
Surgical theatre for laparotomy	☐	☐	☐	☐	☐

Why is surgical theatre for laparotomy not readily available? Please tick all that apply.
**[Opportunity, Physical, and Capability, Physical]*

- ☐ No surgical theatre at hospital
- ☐ No dedicated obstetric theatre at the hospital
- ☐ The surgical theatre is too busy
- ☐ Inconsistent supplies in theatre
- ☐ Incorrect supplies in theatre
- ☐ Inadequate staffing - no anaesthetist
- ☐ Inadequate staffing - no trained surgeon
- ☐ Other (please specify): ____________________

Assistance

What type of assistance would you require if you were asked to do the following to manage a postpartum haemorrhage after a vaginal birth? Please tick all that apply. *
[Capability, Physical, and Opportunity, Physical, and Opportunity, Social]

	More training	More supervision	More supplies	More time	No assistance	Not able to do
Administer ergometrine	☐	☐	☐	☐	☐	☐
Uterine balloon tamponade	☐	☐	☐	☐	☐	☐
Bimanual compression	☐	☐	☐	☐	☐	☐
Administer IV fluids	☐	☐	☐	☐	☐	☐
Internal uterine exploration in theatre	☐	☐	☐	☐	☐	☐
Administer misoprostol	☐	☐	☐	☐	☐	☐
Administer tranexamic acid (TXA)	☐	☐	☐	☐	☐	☐
Administer carbetocin	☐	☐	☐	☐	☐	☐
Manual removal of placenta	☐	☐	☐	☐	☐	☐

What type of assistance would you require if you were asked to do the following to manage a postpartum haemorrhage after a vaginal birth? Please tick all that apply. *
[Capability, Physical, and Opportunity, Physical, and Opportunity, Social]

	More training	More supervision	More supplies	More time	No assistance	Not able to do
Administer oxytocin	☐	☐	☐	☐	☐	☐
Administer carboprost	☐	☐	☐	☐	☐	☐
Uterine massage	☐	☐	☐	☐	☐	☐
Internal examination in delivery room	☐	☐	☐	☐	☐	☐
Blood pressure and pulse check	☐	☐	☐	☐	☐	☐
Admission to ICU	☐	☐	☐	☐	☐	☐
Repair of high vaginal or cervical tears	☐	☐	☐	☐	☐	☐
Non-pneumatic anti-shock garment (NASG)	☐	☐	☐	☐	☐	☐

Multiple Interventions

Imagine that you were asked to perform multiple interventions: e.g. uterine massage, uterotonic drugs, tranexamic acid, IV fluids, examination of the genital tract, in the shortest possible time period. To what extent do you agree that this would...? *

	Strongly disagree	Disagree	Neither agree nor disagree	Agree	Strongly agree	I don't know
Make sense to me *[Capability, Psychological]*	☐	☐	☐	☐	☐	☐
Be too much for one person to do *[Motivation, Reflective]*	☐	☐	☐	☐	☐	☐
Help to improve the management of postpartum haemorrhage for vaginal births *[Motivation, Reflective]*	☐	☐	☐	☐	☐	☐
Be too much for a team to do *[Opportunity, Social]*	☐	☐	☐	☐	☐	☐
Require us to change how we work as a team *[Opportunity, Social]*	☐	☐	☐	☐	☐	☐
Be easy to remember to do *[Capability, Psychological]*	☐	☐	☐	☐	☐	☐
Be burdensome for me to do *[Motivation, Reflective]*	☐	☐	☐	☐	☐	☐
Be more effective than how postpartum haemorrhage is currently managed for vaginal births at our hospital *[Motivation, Reflective]*	☐	☐	☐	☐	☐	☐

What might make it easier to perform multiple interventions together at your hospital? Please tick all that apply. *

- ☐ Improving team working *[Opportunity, Social]*
- ☐ Having reminders *[Opportunity, Social]*
- ☐ Increasing availability of supplies *[Opportunity, Physical]*
- ☐ Having all the required supplies in one place e.g. a box or a trolley *[Opportunity, Physical]*
- ☐ More training *[Capability, Physical]*
- ☐ Having opportunities to practice *[Capability, Physical]*
- ☐ Having plans, protocols or guidelines in place *[Capability, Psychological]*
- ☐ Other (please specify):

Improving the Detection and Management of Postpartum Haemorrhage

Finally, we would like to ask some questions about improving postpartum haemorrhage detection and management after vaginal births in your hospital specifically.

To what extent do you agree with the following statements...? *

	Strongly disagree	Disagree	Neither agree nor disagree	Agree	Strongly agree
Postpartum haemorrhage detection after vaginal birth needs to improve at my hospital *[Motivation, Reflective]*	☐	☐	☐	☐	☐
Postpartum haemorrhage management after vaginal birth needs to improve at my hospital *[Motivation, Reflective]*	☐	☐	☐	☐	☐

Are any of the following strategies currently in place at your hospital to improve postpartum haemorrhage detection and management? Please tick all that you know are in place. *

- ☐ Training and education *[Capability, Physical, and Capability, Psychological]*
- ☐ Decision aids *[Capability, Psychological]*
- ☐ Visual reminders/ posters *[Capability, Psychological]*
- ☐ Feedback on current practice *[Capability, Psychological]*
- ☐ Guidelines and protocols on display *[Opportunity, Physical]*
- ☐ Peer support *[Opportunity, Social]*
- ☐ Local postpartum haemorrhage (PPH) champions and leads *[Opportunity, Social]*
- ☐ Improving ease of access to supplies *[Opportunity, Physical]*

☐ None of these strategies are in place
☐ Other (please specify): ____________________

How effective do you think each of the following strategies might be in improving postpartum haemorrhage detection and management at your hospital? *

	Not at all effective	Slightly effective	Moderately effective	Very effective	Extremely effective	No opinion
Training and education	☐	☐	☐	☐	☐	☐
Decision aids	☐	☐	☐	☐	☐	☐
Reminders e.g. posters	☐	☐	☐	☐	☐	☐
Feedback on current practice	☐	☐	☐	☐	☐	☐
Guidelines and protocols on display	☐	☐	☐	☐	☐	☐
Peer support	☐	☐	☐	☐	☐	☐
Local postpartum haemorrhage (PPH) champions and leads	☐	☐	☐	☐	☐	☐
Improving ease of access to supplies	☐	☐	☐	☐	☐	☐

COVID-19

The final section of this survey will focus on the COVID-19 (coronavirus) pandemic.

Do you think that the COVID-19 pandemic has affected how you detect and manage postpartum haemorrhage? *

☐ Yes - how I **detect** postpartum haemorrhage has changed
☐ Yes - how I **manage** postpartum haemorrhage has changed
☐ Yes - how I **detect and manage** postpartum haemorrhage has changed
☐ No - how I detect and manage postpartum haemorrhage has not changed

How has your practice surrounding the detection and management of postpartum haemorrhage changed? As a result of COVID-19... *

	Strongly disagree	Disagree	Neither agree nor disagree	Agree	Strongly agree
Non-COVID maternal care has been deprioritised *[Motivation, Reflective]*	☐	☐	☐	☐	☐
There is less access to routine tests for PPH *[Opportunity, Physical]*	☐	☐	☐	☐	☐

	Strongly disagree	Disagree	Neither agree nor disagree	Agree	Strongly agree
It is more difficult to access medications to manage PPH *[Opportunity, Physical]*	☐	☐	☐	☐	☐
There are less staff available to monitor women pre-birth *[Opportunity, Social]*	☐	☐	☐	☐	☐
There are less staff available to monitor women post-birth *[Opportunity, Social]*	☐	☐	☐	☐	☐
Patients are coming to hospital less *[Opportunity, Social]*	☐	☐	☐	☐	☐
We have more equipment and supplies to detect and manage PPH *[Opportunity, Physical]*	☐	☐	☐	☐	☐
I have less time to detect and manage PPH *[Opportunity, Physical]*	☐	☐	☐	☐	☐
There are new policies and guidelines to follow *[Capability, Psychological]*	☐	☐	☐	☐	☐
The maternity ward has been converted to a COVID-19 ward *[Opportunity, Physical]*	☐	☐	☐	☐	☐
More women are choosing to have home births than come into the hospital *Opportunity, Social]*	☐	☐	☐	☐	☐

Thank You

Thank you very much for the time you have taken to complete this survey.

If there is anything further you would like to share about anything covered in this survey, please feel free to do so in the box below:
